# A novel image-based neuronal network model framework for understanding visual multistability and neurological disorders

**DOI:** 10.3389/fncom.2026.1793265

**Published:** 2026-04-29

**Authors:** Kaito N. Hikino, Marina Nakayama, Yihui Wu, Victor J. Barranca

**Affiliations:** Department of Mathematics and Statistics, Swarthmore College, Swarthmore, PA, United States

**Keywords:** autism, balanced networks, compressive sensing, multistability, neuronal networks, nonlinear dynamics

## Abstract

While perceptual multistability arises from many types of stimuli across different sensory systems, there are common dynamical features that may be rooted in universal organizing principles underlying perception. We probe the fundamental mechanisms responsible for visual multistability using a neuronal network model framework in which a set of realistic images directly drives competing pools of neurons with nonlinear dynamics. Incorporating balanced network architecture, long-range connections from excitatory neurons to inhibitory neurons in competing pools, and a dynamic spiking threshold, the model produces irregular percept switching and replicates key experimental observations regarding dominance durations in binocular rivalry. Using a sequence of short-time observations of neuronal dynamics, we derive a new methodology for reconstructing the dynamic percept that generalizes to an arbitrary number of percepts, suggesting how rivalry, fusion, and interocular grouping may serve as different states in a single decision-making system. The model dynamics illustrate that perceptual alternations are potentially rooted in the breakdown of balance between excitation and inhibition when the spiking thresholds of suppressed neurons become sufficiently small, with more balanced dynamics generally facilitating longer dominance durations. Finally, we apply our model analysis toward characterizing the causes of psychiatric or neurological disorders, such as amblyopia and autism. Increasing the strength of connections manifesting from the pool of neurons associated with the stronger eye in amblyopia, we find the weaker eye experiences shorter dominance durations as found experimentally, supporting the notion that sufficiently imbalanced inter-eye competition prompts the suppression of information from the monocular stimulus corresponding to the weakened eye. Similarly, we show increasing the ratio of excitatory to inhibitory inputs in the network systematically yields longer dominance durations as observed for individuals with autism, and we thus demonstrate support for the excitation/inhibition imbalance hypothesis for autism.

## 1 Introduction

Human sensory systems are generally optimized for processing natural stimuli encountered when traversing typical environmental settings, where, for example, the sensory inputs into each eye are highly similar (Field, 1994; Kaas, 1989, 2008; Barlow, 1961, 2001). However, sensory signals can instead be ambiguous or artificially generated, yielding multiple reasonable interpretations and thus several possible percepts. In such situations, the percept experienced may irregularly alternate between these feasible interpretations, giving rise to *perceptual multistability*. One notable example is binocular rivalry, which occurs when two highly dissimilar images are presented to each eye; one of the images is randomly perceived at first and then the percept switches to the opposite monocular stimulus, with subsequent perceptual alternations occurring at random (Dutour, 1760; Wheatstone, 1838; Blake and Fox, 1974; Breese, 1909; Wilke et al., 2003). Furthermore, perceptual bistability may occur even when the monocular stimuli are similar, including for the Necker cube with multiple reasonable depth organizations (Necker, 1832) and for situations in which ambiguous structure arises from motion (Ullman, 1979). More generally, multistable rivalry corresponding to more than two percepts is found for a host of stimuli, such as for Gabor patch monocular stimuli with certain orientation differences (Riesen et al., 2019) and for two superimposed drifting gratings (Hupé and Rubin, 2003). Perceptual multistability is prevalent beyond human vision and is also observed in non-human mammals (Sengpiel et al., 1994; Carter et al., 2020), olfaction (Zhou and Chen, 2009), tactile processing (Holcombe and Seizova-Cajic, 2008), and audition (Hupe et al., 2008); despite the differences in stimuli, the commonality in the features of perceptual multistability across its many manifestations suggests a universal theoretical framework may offer rich insights into the phenomenon that may reveal fundamental organizing principles underlying the structure-function relationship in the brain.

Experimental evidence has long underlined a correlation between percepts experienced in binocular rivalry and activity in the primary visual cortex (V1) (Polonsky et al., 2000; Leopold and Logothetis, 1996), but other parts of the visual system and the parietal cortex have been associated with the phenomenon as well (Haynes et al., 2005). The classical mechanistic hypothesis, known as eye-based rivalry, attributes binocular rivalry to competition between two groups of neurons driven by the two respective monocular stimuli. While the early visual system is well known to be composed of neurons with monocular inputs (Blake and Logothetis, 2002) and many experiments indeed provide support for eye-based rivalry (Xu et al., 2016), the dynamics of binocular and feature-selective neurons in the later visual system are increasingly associated with perceptual alternations and indicate rivalry may instead be stimulus-based. Often given as further support for a higher-level and stimulus-based rivalry mechanism, more than two percepts can arise when complementary pieces of two monocular stimuli may be combined into various globally coherent images; this generates perceptual alternations between the actual monocular stimuli and the reasonably regrouped coherent images in what is called interocular grouping (Kovacs et al., 1996; Suzuki and Grabowecky, 2002; Golubitsky et al., 2019; Sterzer et al., 2009). It is possible that these two notions of rivalry together yield a fuller picture of the phenomenon (Lee and Blake, 1999; Tong, 2001), and the theoretical framework we develop seeks to explore the fundamental mechanisms at play across all forms of perceptual multistability and an arbitrary number of competing percepts.

A myriad of detailed theories regarding the necessary and sufficient conditions for binocular rivalry in particular have been put forward over the years, using both experiment-based (Tong, 2001; Levelt, 1965) and model-based settings (Wilson, 2003; Said and Heeger, 2013; Dayan, 1998; Matsuoka, 1984). Across these studies, a combination of internally-generated noise, slow adaptation, and cross-column inhibition are most commonly employed, though a fully unified framework for perceptual multistability and the underlying stimulus encoding principles at play remains an active interdisciplinary subject of investigation (Logothetis et al., 1996; Tong et al., 2006). Mathematical models have been highly successful at replicating important experimental results, but most take the form of either highly idealized rate models that abstract over the dynamics of individual neurons (Shpiro et al., 2009; Li et al., 2017) or utilize stimuli represented by only a single parameter rather than realistic images (Laing and Chow, 2002; Wang et al., 2020).

Addressing these limitations, we develop a large-scale multi-layer network model framework for perceptual multistability that features competing pools of neurons corresponding to potential percepts, which each take the form of realistic images represented by pixel matrices. By deriving and then leveraging an approximate underlying mapping between the network image inputs and recently evoked network activity, the nonlinear dynamics of the individual neurons may then be used to both determine and successfully reconstruct the dynamic percept. Going beyond related previous work, which was limited to binocular rivalry and required long-time activity data collected over the entire period in which each percept was dominant (Barkdoll et al., 2023), we formulate a new percept recovery methodology that requires only the recent history of the network dynamics, comparable to human reaction time. The novel percept recovery framework is able to seamlessly transition between rivalry for an arbitrary number of percepts and fusion, depending on the various individual stimuli utilized. While our two-layer network model may be viewed in some cases as a mechanistic representation of the interplay between the retina and V1 (Barlow, 1981; Spear et al., 1996), it may also be more generally interpreted as a higher-level neuronal system carrying out the selection process among competing potential percepts. We hypothesize that fusion, binocular rivalry, and interocular grouping may therefore all be possible states of a single dynamical system in which many plausible percepts compete, rather than entirely separate and independent processes.

Especially in light of the observation that natural image features modulate rivalrous dynamics in experiments (Alais and Melcher, 2007; Baker and Graf, 2009), we incorporate complex monocular stimuli in the first layer, and provide a modeling framework that is adaptable to any set of image inputs. Similarly, since a growing body of evidence suggests that large excitatory and inhibitory neuronal inputs are dynamically counteracted so input fluctuations yield irregular spiking activity (London et al., 2010; Haider et al., 2006; Miura et al., 2007), we assume the second layer is composed of competing pools of downstream networks that would exhibit such balanced dynamics in isolation, facilitating the stochasticity of the perceptual alternations (van Vreeswijk and Sompolinsky, 1996; Troyer and Miller, 1997). Competition is incorporated via long-range connections from excitatory neurons in one pool to inhibitory neurons in the others, supported by experimental observations demonstrating long-range connections largely manifest from excitatory neurons (Stettler et al., 2002; Douglas and Martin, 2004; Tamamaki and Tomioka, 2010). Finally, spike-frequency adaptation is included via a dynamic spiking threshold (Freeman, 2005; Kilpatrick and Bressloff, 2010), providing a fatigue mechanism that promotes the switch between which pool of neurons is dominant. Each downstream pool corresponds to a different attractor in a decision-making system, and when the network-averaged firing rate of one pool is significantly larger than the competing pools over a given span of time, the percept corresponding to the more active pool is chosen.

In support of our modeling framework, we produce periods of dominance comparable to those found in experiments and demonstrate agreement with Levelt's laws, which are often used as a proof-of-concept for mathematical models of binocular rivalry (Levelt, 1965). We then apply a combination of model analysis and systematic investigation of the model dynamics to better characterize the precise neuronal network activity responsible for the switch in dominance, revealing that a breakdown in balanced dynamics may occur in the vicinity of an alternation. By adjusting the heterogeneity in the feedforward inputs and measuring the resultant dominance durations, we similarly provide evidence that networks with more balance between excitation and inhibition have longer dominance durations.

Given that numerous experimental studies indicate neurological or psychiatric disorders stem from abnormal brain connectivity (Gao and Penzes, 2015; Nelson and Valakh, 2015; Rosenberg et al., 2015) and recent studies suggest a relationship between perceptual multistability and neurological disorders, our general model framework provides a natural context for testing such theories. Amblyopia, a disorder in which the monocular stimulus information from only a single eye becomes primarily used in visual perception, is a leading cause of vision loss among children and can arise for a number of reasons, including improper eye alignment, imbalanced muscular structure, and substantially different refractive power among the two eyes (Attebo et al., 1998). Across these physiological causes, the result is that the brain ultimately suppresses much of the information provided by the weakened (amblyopic) eye and a potential mechanism for such suppression is an asymmetry in the strength of the long-range connections between monocular neurons, which mediate competition in our model framework. We show that by increasing the disparity in the strength of long-range connections originating from two pools of downstream neurons in our model, the dominance durations corresponding to the amblyopic eye indeed become relatively small, giving credence to the hypothesized mechanism for amblyopia akin to competition in binocular rivalry. In a similar vein, since individuals with autism exhibit longer dominance durations in binocular rivalry (Robertson et al., 2013; Spiegel et al., 2019), we are able to use our model to test the excitation/inhibition (E/I) hypothesis that an elevated ratio of excitatory to inhibitory neuronal inputs may be responsible for autism (Gao and Penzes, 2015; Nelson and Valakh, 2015; Rosenberg et al., 2015). In particular, by increasing the strength of connections manifesting from excitatory neurons or decreasing the strength of connections arising from inhibitory neurons, we observe lengthening dominance durations as found for individuals with increasingly acute autism. We further demonstrate that these changes to the network model indeed facilitate imbalanced dynamics and elevated spiking thresholds, supporting the E/I hypothesis and the feasibility of both potential pathways toward imbalance. Together, this suggests dominance durations in binocular rivalry may serve as a noninvasive indicator of autism and that adjusting the balance of excitation and inhibition in the brain may potentially offset the conditions found in severe autism.

## 2 Methods

### 2.1 Neuronal network model for perceptual multistability

To study perceptual multistability in the case of an arbitrary number of competing percepts, we consider a two-layer network model with *q* competing pools of *N* downstream neurons, which are each forced by their respective image inputs, *p*_*y*_ for *y* = 1, …, *q*. Each pool contains a population of *N*_*E*_ excitatory neurons and a population of *N*_*I*_ inhibitory neurons (subscripts *k* = *E* and *k* = *I* denote excitatory and inhibitory neurons, respectively), with each neuron governed by pulse-coupled integrate-and-fire (I&F) dynamics. Incorporating more detailed spiking dynamics than firing rate models while maintaining computational feasibility, the I&F model is commonly used in large-scale neuronal network models and includes the key nonlinear features of neuronal voltage dynamics (Mather et al., 2009; Rauch et al., 2003; Barranca et al., 2014a; Rangan and Cai, 2006; Burkitt, 2006; Abbott, 1999). The *i*^th^ neuron in the *k*^th^ population of the *x*^th^ downstream pool in the network has voltage, ${v_{x}}_{k}^{i}$, governed by the system of differential equations


$$\begin{array}{l} \frac{dv_{x_{k}}^{i}}{dt}=-g_{L}\left(v_{x}^{i}-V^{Re}\right)+\sum_{\begin{array}{c} j=1\\ j\neq i \end{array}}^{N_{E}}R_{xkE}^{ij}\sum_{h}\delta \left(t-\tau_{xE}^{jh}\right)\\ +\sum_{\begin{array}{c} j=1\\ j\neq i \end{array}}^{N_{I}}R_{x_{kI}}^{ij}\sum_{h}\delta \left(t-\tau_{x_{I}}^{jh}\right)+\sum_{\begin{array}{c} y=1\\ y\neq x \end{array}}^{q}\sum_{\begin{array}{c} j=1\\ j\neq i \end{array}}^{N_{E}}C_{x_{kE}}^{yij}\sum_{h}\delta \left(t-\tau_{y_{E}}^{jh}\right)\\ +\sum_{j}F_{x}^{ij}p_{x}^{j}, \end{array}$$ (1a)

$$\begin{array}{lll} \frac{d{\theta_{x}}_{k}^{i}}{dt} & = & -\lambda \left({\theta_{x}}_{k}^{i}-\theta_{k}\right), \end{array}$$ (1b)

and evolves from reset potential, *V*^*Re*^, until reaching its dynamic firing threshold, ${\theta_{x}}_{k}^{i}$, when the neuron consequently fires. At this time, its voltage is instantaneously reset to *V*^*Re*^ and the voltages of all post-connected neighbors are instantaneously altered upon integrating over the Dirac delta functions, δ(·), in Equation 1a. The spike times for the *i*^th^ neuron in the *k*^th^ population of the *x*^th^ pool are given by ${\tau_{x}}_{k}^{ih}$ and are indexed by *h* = 1, 2, …  in ordering the spike times.

The *N*×*N* recurrent connectivity matrix for the *x*^th^ downstream pool, ***R*_*x*_**, dictates the neuronal interactions within the pool and is indexed such that ${R_{x}}_{kl}^{ij}$ gives the interaction strength between the *i*^th^ post-connected neuron in the *k*^th^ population and the *j*^th^ pre-connected neuron in the *l*^th^ population. Every entry of the recurrent connectivity matrix is determined by a Bernoulli distribution, where ${R_{x}}_{kl}^{ij}=R_{kl}/\sqrt{K}$ with probability *K*/*N*_*l*_ and ${R_{x}}_{kl}^{ij}=0$ otherwise. As a result, a downstream neuron is post-connected to *K* excitatory and *K* inhibitory neurons within its pool on average, where the excitatory connection strength *R*_*kE*_>0 and inhibitory connection strength *R*_*kI*_ < 0. The recurrent connectivity is assumed sparse in this model, as typically observed in experiment (Ganmor et al., 2011; He et al., 2007), implying 1≪*K*≪*N*_*E*_, *N*_*I*_.

Since each pool of neurons reflects preference for a particular choice of percept, a neuron in the *x*^th^ downstream pool receives external forcing from its corresponding input image, *p*_*x*_, which is mediated by a feedforward connectivity matrix, ***F*_*x*_**. The constant input image vector is gray-scale with *n* = 10*N*_*E*_ total pixel entries, reflecting the large number of input components relative to downstream neurons in common compressive layers across sensory systems. However, we have verified that a larger number of downstream neurons may be utilized without loss of generality in this study. The feedforward connectivity submatrix, _***F*_*x*_*k***_, determines the connections between the upstream and downstream layers such that ${F_{x}}_{k}^{ij}$ denotes the feedforward connection strength between the *j*^th^ component of the stimulus vector, ${p_{x}}^{j}$, and the *i*^th^ neuron in the *k*^th^ population of the *x*^th^ pool. Considering a hierarchy of several network layers may all be partially responsible for various aspects of perceptual multistability (Wilson, 2003), we incorporate only broad shared characteristics in our network connectivity to isolate the central mechanistic contributing factors.

Since receptive field structure in feedforward connectivity is common in many sensory systems, where downstream neurons are typically most stimulated by a range of stimuli with similar characteristics (Wilson, 2001; Welker, 1976; Wiesel, 1960), we incorporate the *spatially localized* nature of these receptive fields for additional realism while still maintaining generality (Barranca and Zhu, 2018). To include spatial structure, a pixel in *n*-vector input ***p*_*z*_** is mapped to a distinct location with integer coordinates on a $\left[1,\;\sqrt{n}\right]\times \left[1,\;\sqrt{n}\right]$ Cartesian grid corresponding to its row and column location in the pixel matrix that generated vectorization ***p*_*z*_**. Each row of the corresponding feedforward connectivity matrix is then given a random location on this grid, such that the receptive field of the *i*^th^ downstream neuron in the pool is centered at (*x*_*i*_, *y*_*i*_). Incorporating a mix of spatial localization and randomness, we assume the probability, *P*, that the *i*^th^ downstream neuron samples an input pixel with spatial coordinates (*x*_*j*_, *y*_*j*_) is given by


$$\begin{array}{l} P=\rho \exp\left(-\left[{\left(x_{i}-x_{j}\right)}^{2}+{\left(y_{i}-y_{j}\right)}^{2}\right]/2\sigma^{2}\right), \end{array}$$ (2)

where ρ determines the sampling probability if (*x*_*i*_, *y*_*i*_) = (*x*_*j*_, *y*_*j*_), when the receptive field is centered at the location of a given pixel, and σ determines the receptive field size. We use a (ρ, σ) parameter choice shown previously to well encode a single 100 × 100 pixel natural scene stimulus with *N* samples (Barranca and Zhu, 2018), namely σ = 1.75 pixels and ρ = 0.96 in Equation 2, which yields moderately-sized receptive fields. The external drive into a downstream neuron in the *k*^th^ population of a given pool is normalized to have expected value $\sqrt{K}f_{k}m_{0}$, where *m*_0_ and *f*_*k*_ are O(1) parameters applying to all pools. Note that *m*_0_ determines the overall external drive strength for the two populations within a given pool, while *f*_*k*_ specifically determines the external drive strength for the *k*^th^ population only. For each population, stimuli sampled in this manner drive the *nonlinear* (I&F) network dynamics that ultimately are decoded to obtain the time-varying percept.

**Figure 1:**
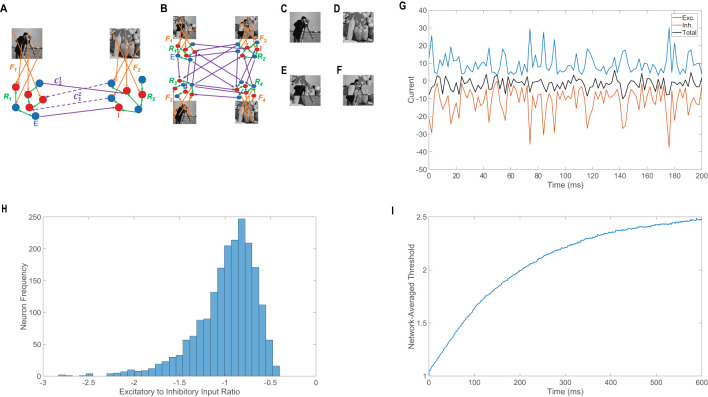
Main features of the neuronal network model of perceptual multistability. **(A)** Schematic of the two-layer neuronal network model for binocular rivalry. Pool 1 and pool 2 are composed of downstream I&F neurons driven by different image inputs via feedforward connectivity matrices, *F*_1_ and *F*_2_, respectively (orange). Recurrent connectivity matrices, *R*_1_ and *R*_2_, govern interactions between neurons within pool 1 and within pool 2, respectively (green). Long-range connections between neurons in the two pools (purple) go from excitatory neurons (blue, *E*) to inhibitory neurons (red, *I*). **(B)** Schematic of the network model with four competing percepts, akin to interocular grouping. **(C–F)** 100 × 100 gray-scale images that are vectorized to produce image inputs in competing pools of neurons. Images **(E, F)** are complementary rearranged versions of **(C, D)**. **(G)** Dynamics of the excitatory (blue), inhibitory (red), and total (black) current inputs in a sample excitatory neuron in a balanced pool. **(H)** Histogram depicting the ratio of total excitatory input to total inhibitory input across neurons in a balanced pool. **(G, H)** consider the dynamics of an isolated downstream pool in the model prescribed by schematic A in the absence of both long-range connections and adaptation. **(I)** Population-averaged threshold dynamics for excitatory neurons in an isolated pool in a balanced network incorporating spike-frequency adaptation. Parameters utilized are *R*_*EE*_ = *R*_*IE*_ = 1, *R*_*II*_ = −1.8, *R*_*EI*_ = −2, *N*_*E*_ = 1, 000, *N*_*I*_ = 1, 000, *f*_*E*_ = 1, *f*_*I*_ = 0.8, *m*_0_ = 0.4, *K* = 40, λ = 0.05, ϕ = 0.3, θ_*E*_ = 1, and θ_*I*_ = 0.8. The voltages and thresholds are nondimensionalized such that *V*^*Re*^ = 0 and $g_{L}=50{\text{s}}^{-1}$, corresponding to the conventional voltage time-scale of 20ms (McLaughlin et al., 2000; Barranca et al., 2014b; Brette et al., 2007).

Competition between the percepts is mediated by long-range connections from excitatory neurons in one pool to inhibitory neurons in the other pools. The long-range connections are given by matrix $C_{x}^{y}$, where ${C_{x}^{y}}_{kl}^{ij}$ denotes the long-range connection strength between the *i*^th^ post-connected neuron in the *k*^th^ population of the *x*^th^ pool and the *j*^th^ pre-connected neuron in the *l*^th^ population of the *y*^th^ pool. The entries of $C_{x}^{y}$ are prescribed like those of the recurrent connectivity matrices, with the main exception being that the only nonzero long-range connections possible are from excitatory to inhibitory neurons, meaning ${C_{x}^{y}}_{IE}^{ij}=R_{IE}/\sqrt{K}$ with probability *K*/*N*_*E*_ and ${C_{x}^{y}}_{kl}^{ij}=0$ otherwise. As a result, when one pool is in a high-firing state, it sends a large number of excitatory impulses to inhibitory neurons in the competing pools and thus suppresses the other potential percepts. With all of the major structural features in place, the architecture of the modeling framework is represented graphically in Figure 1A for binocular rivalry and in Figure 1B for rivalry between four percepts, as found in interocular grouping experiments. Representative model image inputs utilized, ***p*_*x*_**, are depicted in Figures 1C–F. Model parameters are also listed in the caption of Figure 1.

Since the recurrent connectivity strength parameters, *R*_*kl*_, are all O(1), if the dominant pool receives only weak competitive drive from the suppressed pools, then the average excitatory and inhibitory inputs into each downstream neuron in the dominant pool are of the same order and intermittent fluctuations in neuronal input are primarily responsible for the irregular firing events. This produces an approximately constant level of asynchronous activity across neurons in the dominant pool throughout most of a dominance duration (van Vreeswijk and Sompolinsky, 1996; Barranca et al., 2019). Note that we utilize a balanced network configuration within each pool in isolation (van Vreeswijk and Sompolinsky, 1996; Miura et al., 2007; Barranca et al., 2019; Barranca and Zhou, 2019), which yields the asynchronous activity in the dominant pool that is largely responsible for irregularly breaking symmetry to facilitate a new percept and is specifically leveraged in our derivation of the network input-output mapping used in percept recovery. Though alternative mechanisms for generating the stochasticity in dominance durations have been previously used in the study of binocular rivalry and often directly incorporate noise (Freeman, 2005), balanced networks have recently yielded dynamics that agree with key experimental results while making the more realistic assumption of internally-generated irregularity (Wang et al., 2020; Barkdoll et al., 2023).

For a sample downstream neuron in an isolated pool with balanced dynamics, we plot in Figure 1G the time dynamics of its total excitatory and inhibitory inputs, which dynamically cancel and are only sporadically large enough in sum for a spike to occur. This is primarily true across the whole balanced network, and thus only a relatively constant small proportion of neurons typically spikes in a given small time window. The time-averaged ratio between the total excitatory and total inhibitory input into a given neuron (E/I ratio) is primarily near −1 across the network, as shown in Figure 1H, demonstrating that the excitatory and inhibitory inputs are largely proportional on a neuron-by-neuron basis. We note an E/I ratio closer to 0 implies an excess of inhibitory input and an E/I ratio less than −1 implies excessive excitation, but E/I ratios far from −1 are relatively rare in a balanced network.

Without a slow fatigue mechanism, the system would exhibit winner-take-all behavior rather than multistability, and modeling frameworks typically address this by including either synaptic depression or spike-frequency adaptation (Freeman, 2005; Kilpatrick and Bressloff, 2010). Since these mechanisms both diminish the excitability of the dominant pool upon sufficiently sustained firing activity, we incorporate spike-frequency adaptation into our model via a dynamic firing threshold for concreteness and analytical tractability (Brown and Adams, 1980; Benda and Herz, 2003; Barranca et al., 2014a). As a result, the firing threshold of the *i*^th^ neuron in the *k*^th^ population of the *x*^th^ downstream pool, ${\theta_{x}}_{k}^{i}\left(t\right)$, increases by positive constant ϕ at the instant that neuron fires. Several spikes occurring in rapid succession produce an accumulated increase in threshold, requiring more excitation for that neuron to fire. Between firing events for this neuron, ${\theta_{x}}_{k}^{i}\left(t\right)$ is governed by Equation 1b, such that its dynamic firing threshold decays at rate λ to the fixed non-adapted threshold for all neurons in the *k*^th^ population, θ_*k*_. As the firing thresholds of neurons in a recently suppressed pool decrease in the absence of many spikes, the overall firing activity of the suppressed pool eventually increases with time and offers the opportunity for a new percept to dominate.

In Figure 1I, we plot the population-averaged threshold dynamics for the excitatory neurons in a given pool in isolation, which demonstrates a relatively rapid initial ascent followed by saturation in the long-time limit. As a result, the proportion of excitatory neurons firing decreases in time at first and then stabilizes around a decreased mean with minor fluctuations. Previous theoretical work shows balanced dynamics with diminished overall activity still persist in the presence of moderate adaptation, and when adaptation is exclusively present in only the excitatory neurons, the parameter regime corresponding to balanced dynamics enlarges (Barranca et al., 2019). As a whole, the internally-generated irregularity from the balanced network architecture and the dynamic firing threshold together contribute to the randomness in dominance durations, and the long-range connections from excitatory to inhibitory neurons mediate sustained competition between pools, providing the fundamental ingredients for multistability.

### 2.2 Mapping between network inputs and evoked dynamics

**Figure 2:**
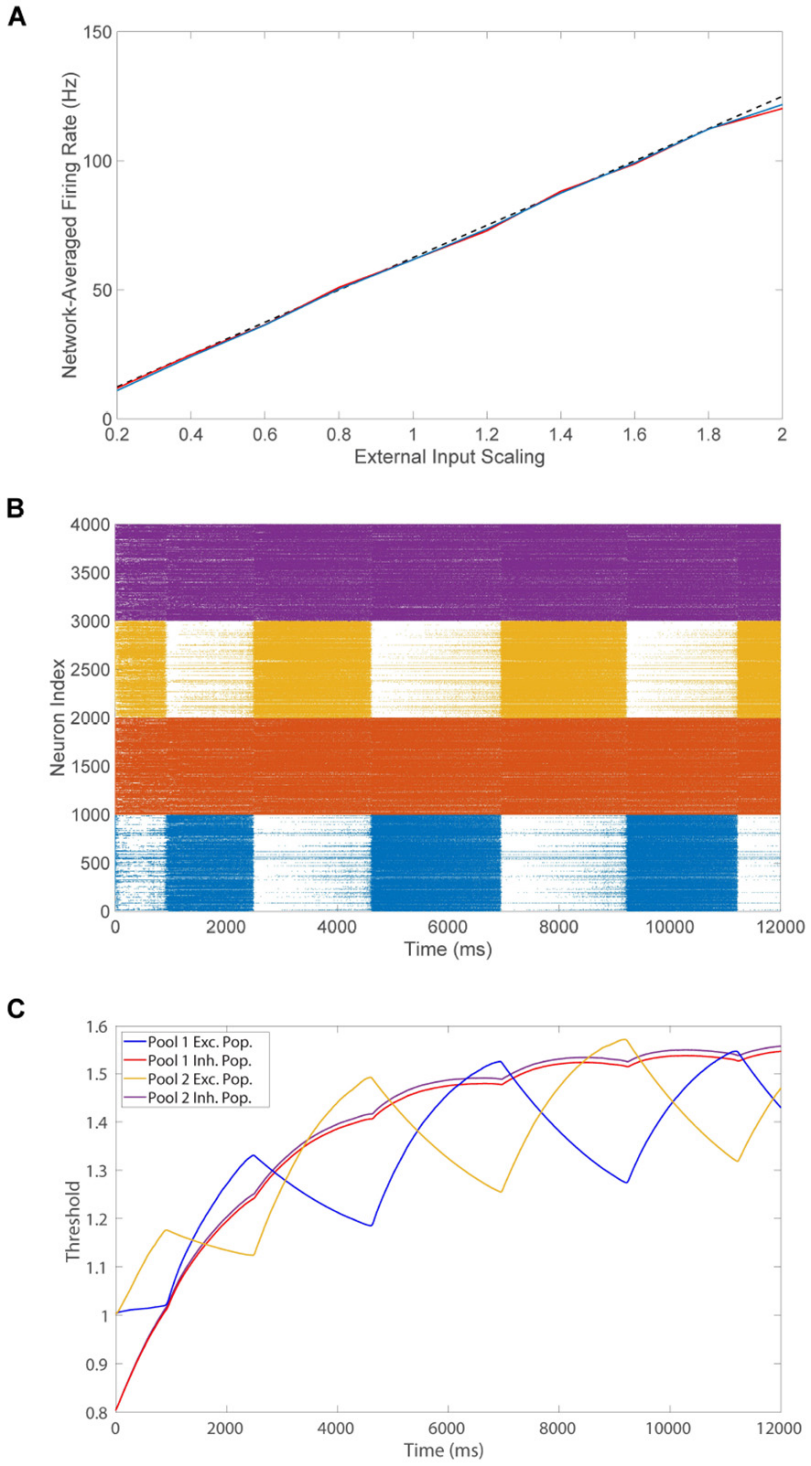
Multistability and dynamics of the network model. **(A)** Population-averaged firing rates across excitatory neurons (blue) and inhibitory neurons (red) in a model network pool as a function of the external input strength scaling. The theoretical firing rate from Equation 7 is also depicted (dashed) for comparison. **(B)** Raster plot exhibiting the firing times of downstream neurons in a model of binocular rivalry with two competing pools. Neurons in pool 1 are indexed 1 − 2000 (excitatory in blue, inhibitory in red) and neurons in pool 2 are indexed 2001 − 4000 (excitatory in orange, inhibitory in purple). **(C)** Dynamics of the population-averaged firing threshold across all neurons in the binocular rivalry model using the same color scheme and simulation as **(B)**. Parameters are given in Figure 1, for which the excitatory and inhibitory populations theoretically possess the same average firing rates in Equation 7. Additionally, λ = 0.00625 and ϕ = 0.005.

With the aim of predicting as well as accurately recovering the alternating percept, we derive a linear mapping between the image inputs and evoked dynamics across an arbitrary number of competing pools. While the voltage dynamics of neurons on an individual level are nonlinear, the network-averaged firing rate response of an isolated balanced network scales linearly with the network external input strength (van Vreeswijk and Sompolinsky, 1996; Troyer and Miller, 1997; Barranca, 2022). To demonstrate this, in Figure 2A, we increase the overall scaling strength *m*_0_ of the external input for an isolated balanced pool of neurons and depict the population-averaged firing rates in each case. As the external drive strength is increased, the population-averaged firing rate for both the excitatory and inhibitory populations linearly increases, suggesting the existence of a linear input-output mapping. Our derivation is rooted in a coarse-graining approach that leverages probabilistic arguments based on the characteristics of balanced network dynamics to approximate the long-time expected voltage for an arbitrary *i*^th^ neuron in the *k*^th^ population of the *x*^th^ downstream pool in the network, which is described in detail below.

Since the voltage of each spiking neuron is reset to *V*^*Re*^, we consider Equation 1 with initial condition ${v_{x}}_{k}^{i}\left(t=0\right)=V^{Re}$ for both *k* = *E, I* and first seek to approximate both the recurrent and feedforward inputs into a given downstream neuron. In computing the expected recurrent input, we observe that a given neuron integrates a spike train generated by the firing activity of a large number of neighboring neurons and since the spike times of neurons in the balanced regime are weakly correlated, the spike-train input approaches a Poisson point process (Cinlar, 1972). Making this approximation, the subthreshold solution to the initial value problem is


$$\begin{array}{l} v_{x}^{i}(t)=V^{Re}+\sum_{\begin{array}{c} j=1\\ j\neq i \end{array}}^{N_{E}}R_{x_{kE}}^{ij}\Psi_{x_{E}}^{j}(t)+\sum_{\begin{array}{c} j=1\\ j\neq i \end{array}}^{N_{I}}R_{x_{kI}}^{ij}\Psi_{x_{I}}^{j}(t)\\ +\sum_{\begin{array}{c} y=1\\ y\neq x \end{array}}^{q}\sum_{\begin{array}{c} j=1\\ j\neq i \end{array}}^{N_{E}}C_{x_{kE}}^{yij}\Psi_{yE}^{j}(t)+\left(\frac{1-e^{-g_{L}t}}{g_{L}}\right){\sum_{j}F_{x}}^{ij}p_{x}^{j} \end{array}$$ (3a)

$$\begin{array}{l} \Psi_{y_{l}}^{j}(t)=\sum_{s=1}^{T_{y_{l}}^{j}(t)}e^{-g_{L}\left(t-U_{y_{l,s}}^{j}(t)\right)}, \end{array}$$ (3b)

where ${R_{x}}_{kl}^{ij}\cdot {{\text{Ψ}}_{x}}_{l}^{j}\left(t\right)$ approximates the recurrent input from the *j*^th^ neuron of the *l*^th^ population in the *x*^th^ pool into the *i*^th^ neuron of the *k*^th^ population in the *x*^th^ pool at time *t*.

In general, the number of spikes dispatched by the *j*^th^ neuron of the *l*^th^ population in the *y*^th^ pool up to time *t* is approximated by ${T_{y}}_{l}^{j}\left(t\right)$, which is Poisson distributed with expected number of events, ${m_{y}}_{l}^{j}t$, determined by the long-time mean firing rate of the *j*^th^ neuron of the *l*^th^ population in the *y*^th^ pool, ${m_{y}}_{l}^{j}$. In estimating the time of these spikes, the spike train input from the *j*^th^ neuron of the *l*^th^ population in the *y*^th^ pool has spike times denoted by ${U_{y}}_{l,s}^{j}\left(t\right)$, for *s* = 1, 2, … , which are assumed to be distributed uniformly within interval [0, *t*] based on the irregularity of firing events in the balanced regime. Since the probability density function for random variable ${U_{y}}_{l,s}^{j}\left(t\right)$ is $P_{{U_{y}}_{l,s}^{j}\left(t\right)}\left(u\right)=1/t$ in its support, the decaying drive from a past spike in Equation 3 given by random variable ${\Xi_{y}}_{l,s}^{j}\left(t\right)=e^{-g_{L}\left(t-{U_{y}}_{l,s}^{j}\left(t\right)\right)}$ has probability density function $P_{{\Xi_{y}}_{l,s}^{j}\left(t\right)}\left(\xi \right)=1/\left(g_{L}t\xi \right)$ for $\xi \in \left[e^{-g_{L}t},1\right]$ and expected value $\left(1-e^{-g_{L}t}\right)/\left(g_{L}t\right)$. Therefore, in total, ${{\text{Ψ}}_{y}}_{l}^{j}\left(t\right)$ is a sum of approximately independent identically distributed random variables and has expected value $\frac{1-e^{-g_{L}t}}{g_{L}t}{m_{y}}_{l}^{j}t$ over time duration *t*.

Under these approximations and assuming the neuron has not yet fired, the expected subthreshold voltage of the *i*^th^ neuron of the *k*^th^ population in the *x*^th^ pool at time *t* is


$$\begin{array}{l} {\tilde{v}}_{x}^{i}(t)=V^{Re}+\frac{1-e^{-g_{L}t}}{g_{L}}\cdot (\sum_{j}F_{x}^{ij}p_{x}^{j}+\sum_{\begin{array}{c} j=1\\ j\neq i \end{array}}^{N_{E}}R_{xkE}^{ij}m_{x_{E}}^{j}\\ +\sum_{\begin{array}{c} j=1\\ j\neq i \end{array}}^{N_{I}}R_{x}^{ij}m_{x_{I}}^{j}+\sum_{\begin{array}{c} y=1\\ y\neq x \end{array}}^{q}\sum_{\begin{array}{c} j=1\\ j\neq i \end{array}}^{N_{E}}C_{xkE}^{yij}m_{yE}^{j}). \end{array}$$

As *t* → ∞, ${\tilde{v_{x}}}_{k}^{i}\left(t\right)$ approaches


$$\begin{array}{l} {\tilde{v}}_{xk}^{i}=V^{Re}+\frac{1}{g_{L}}\cdot (\sum_{j}F_{x}^{ij}p_{x}^{j}+\sum_{\begin{array}{c} j=1\\ j\neq i \end{array}}^{N_{E}}R_{xk}^{ij}m_{x_{E}}^{j}\\ +\sum_{\begin{array}{c} j=1\\ j\neq i \end{array}}^{N_{I}}R_{xI}^{ij}m_{x_{I}}^{j}+\sum_{\begin{array}{c} y=1\\ y\neq x \end{array}}^{q}\sum_{\begin{array}{c} j=1\\ j\neq i \end{array}}^{N_{E}}C_{x_{kE}}^{yij}m_{yE}^{j}). \end{array}$$

The expected voltage assuming this neuron itself undergoes firing events, ${\bar{v_{x}}}_{k}^{i}$, will be lower than ${\tilde{v_{x}}}_{k}^{i}$ because the voltage is instantaneously reset to *V*^*Re*^ each time it reaches the spiking threshold ${\theta_{x}}_{k}^{i}$. Consequently, since the neuron is expected to spike at rate ${m_{x}}_{k}^{i}$ and the change in voltage at an action potential is $-\left({\theta_{x}}_{k}^{i}-V^{Re}\right)$, the long-time expected voltage for the *i*^th^ neuron in the *k*^th^ population of the *x*^th^ pool is approximately


$$\begin{array}{l} {\bar{v}}_{x}^{i}=V^{Re}+\frac{1}{g_{L}}\cdot (\sum_{j}F_{x}^{ij}p_{x}^{j}+\sum_{\begin{array}{c} j=1\\ j\neq i \end{array}}^{N_{E}}R_{xkE}^{ij}m_{x_{E}}^{j}\\ +\sum_{\begin{array}{c} j=1\\ j\neq i \end{array}}^{N_{I}}R_{x_{kI}}^{ij}m_{x_{I}}^{j}+\sum_{\begin{array}{c} y=1\\ y\neq x \end{array}}^{q}\sum_{\begin{array}{c} j=1\\ j\neq i \end{array}}^{N_{E}}C_{x_{kE}}^{yij}m_{y_{E}}^{j}-m_{xk}^{i}\left({\bar{\theta }}_{xk}^{i}-V^{Re}\right)), \end{array}$$ (4)

where ${\bar{\theta_{x}}}_{k}^{i}$ is the time-averaged firing threshold for the *i*^th^ neuron in the *k*^th^ population of the *x*^th^ pool.

Equation 4 relates the input image driving a downstream neuron in the *x*^th^ pool, ***p*_*x*_**, to the output firing rates and expected voltages across the downstream neurons in that pool, ***m*_*x*_** and ${\bar{v}}_{x}$, respectively. In this sense, we view this as the linear input-output mapping that we will use to determine the dynamic percept and it may be compactly expressed in matrix form as


$$\begin{array}{l} {\bar{V}}_{x}=V^{Re}+\frac{1}{g_{L}}\left(F_{x}p_{x}+R_{x}m_{x}+\sum_{\begin{array}{c} y=1\\ y\neq x \end{array}}^{q}C_{x}^{y}m_{y}-m_{x}\left({\bar{\theta }}_{x}-V^{Re}\right)\right) \end{array}$$ (5)

where the elements are ordered so the first *N*_*E*_ rows (columns) correspond to the *N*_*E*_ excitatory neurons and the next *N*_*I*_ rows (columns) correspond to the *N*_*I*_ inhibitory neurons in the *x*^th^ pool.

Note that the time-averaged threshold of each neuron in the *x*^th^ pool, ${\bar{\theta }}_{x}$, reflects the impact of spike-frequency adaptation and that this mapping applies when the *x*^th^ pool is dominant. This map assumes the dynamical statistics, m_x_, ${\bar{v}}_{x}$, and ${\bar{\theta }}_{x}$, accurately approximate long-time averages, which is expected by the near stationarity of the balanced regime after the adaptation saturates. For the purpose of percept recovery, statistics regarding the network dynamics are computed separately over various time bins commensurate with human reaction time, and then we separately use these datasets to reconstruct each corresponding percept.

Assuming the excitatory neurons in the suppressed pools are nearly silent, such that the cross-pool inputs in Equation 4 have little impact on the expected voltage of a neuron in the dominant pool, we obtain the classical balance conditions for the dominant pool and approximate the population-averaged firing rates for the constituent excitatory and inhibitory populations (van Vreeswijk and Sompolinsky, 1996; Barranca et al., 2019). At the population level, since each neuron in the *l*^th^ population of the *x*^th^ pool is expected to fire at the long-time population-averaged firing rate _*m*_*x*_*l*_ and each neuron in the *k*^th^ population of the *x*^th^ pool is expected to receive *K* incoming recurrent connections from the *l*^th^ population of the *x*^th^ pool with individual connection strength $R_{kl}/\sqrt{K}$, the expected total recurrent input from the *l*^th^ population in the *x*^th^ pool into a neuron in the *k*^th^ population of the *x*^th^ pool is $R_{kl}{m_{x}}_{l}\sqrt{K}$. As a result, taking the average of Equation 4 over all network realizations, we approximate the expected offset in voltage from *V*^*Re*^ as a result of recurrent as well as feedforward inputs for a downstream neuron in the *k*^th^ population of the *x*^th^ pool as


$$\begin{array}{l} d_{xk}=\sqrt{K}\frac{1}{g_{L}}\left(f_{k}m_{0}+R_{kE}m_{xE}+R_{kI}m_{xI}\right). \end{array}$$ (6)

Since the expected voltage and firing rate for a given downstream neuron must remain finite as *K* → ∞ in the large-network limit for asynchronous and irregular dynamics, it is required that *f*_*k*_*m*_0_+*R*_*kE*__*m*_*x*_*E*_+*R*_*kI*__*m*_*x*_*I*_ is $O\left(1/\sqrt{K}\right)$ and approaches zero as *K* → ∞ (van Vreeswijk and Sompolinsky, 1998). As a result, we obtain a system of two linear equations with solution prescribing the population-averaged firing rates for the excitatory and inhibitory populations composing the *x*^th^ pool during its period of dominance


$$\begin{array}{l} m_{xE}=\frac{\left|R_{II}\right|f_{E}-\left|R_{EI}\right|f_{I}}{R_{IE}\left|R_{EI}\right|-R_{EE}\left|R_{II}\right|}m_{0} \end{array}$$ (7a)

$$\begin{array}{l} m_{xI}=\frac{R_{IE}f_{E}-R_{EE}f_{I}}{R_{IE}\left|R_{EI}\right|-R_{EE}\left|R_{II}\right|}m_{0}, \end{array}$$ (7b)

which demonstrates the theoretical linear scaling of the population-averaged firing rates with the feedforward drive strength parameter *m*_0_ as depicted by the theoretical gain curve in Figure 2A. These predictions well agree with the empirical results plotted, confirming the accuracy of our approximations even for a finite network realization.

For the excitatory and inhibitory population-averaged firing rates in Equation 7 to be nonnegative and finite in the large-network limit, the following balance conditions for the connectivity parameters must hold,


$$\begin{array}{l} \frac{f_{E}}{f_{I}}>\frac{\left|R_{EI}\right|}{\left|R_{II}\right|}>\frac{R_{EE}}{R_{IE}}. \end{array}$$ (8)

While conventional balanced network theory considered the case of constant and homogeneous excitatory external inputs driving the two populations in a single pool (van Vreeswijk and Sompolinsky, 1996), we note that our network model framework uses excitatory external input vectors that are heterogeneous and determined by the competing image inputs driving the network dynamics. However, assuming the external input strength scalings for the excitatory and inhibitory populations obey *f*_*E*_>*f*_*I*_ for these heterogeneous inputs, the balance conditions in Equation 8 hold on average and we observe that balanced dynamics are indeed well preserved in the dominant pool.

### 2.3 Recovering percepts from model network dynamics

In order to apply input-output mapping (Equation 5) and recover the percept given measurements of recent network dynamics, we assume the network inputs are unknown and all remaining terms are either given or estimated. In particular, the connectivity matrices are known given a particular network realization and model parameters, *g*_*L*_ and *V*^*Re*^, are also known. Equation 5 thus takes the form of a linear system with unknown p_x_, particularly when expressed as


$$\begin{array}{l} {\text{F}}_{\text{x}}{\text{p}}_{\text{x}}=g_{L}\left({\bar{\text{v}}}_{\text{x}}-{\text{V}}^{\text{Re}}\right)-{\text{R}}_{\text{x}}\;{\text{m}}_{\text{x}}-\sum_{\begin{array}{c} y=1\\ y\neq x \end{array}}^{q}{\text{C}}_{\text{x}}^{\text{y}}\;{\text{m}}_{\text{y}}+{\text{m}}_{\text{x}}\left({\bar{\theta }}_{\text{x}}-{\text{V}}^{\text{Re}}\right). \end{array}$$ (9)

Considering feedforward pathways in the brain are likely optimized for efficient coding and often compressive, in that there are many more upstream neurons than downstream neurons (Barlow, 1981; Barranca et al., 2014b; Welker, 1976; Mori et al., 1999; Knudsen and Konishi, 1978; Brecht and Sakmann, 2002), Equation 9 may take the form of a highly underdetermined linear system with infinitely many solutions. Therefore, to accurately choose the percept most closely aligned with the network dynamics, we leverage compressive sensing (CS) theory, a recent signal processing advance that provides a means of efficiently sampling and reconstructing signals which are sparse in a domain of interest (Candes et al., 2006; Candes and Wakin, 2008; Donoho, 2006). Since natural scenes and gratings, which are often used as stimuli in visual multistability, are known to have sparse representations in frequency-based domains (Field, 1994), we posit that CS techniques are suitable in their recovery. As CS-based reconstructions typically necessitate linear measurements of constant sampled data (Candes et al., 2006; Donoho, 2006) and our model instead exhibits nonlinear time dynamics, the derived input-output map in Equation 9 provides a means of addressing this potential limitation.

*The percept recovery framework is as follows:* To estimate the necessary dynamical statistics in Equation 9, we first collect the network model output over a fixed small time window, comparable to human reaction time, for each of the *q* pools of downstream neurons. For a given time window, we record the firing rates, time-averaged voltages, and time-averaged thresholds across all downstream neurons to obtain estimates for ***m*_*x*_**, ${\bar{v}}_{x}$, and ${\bar{\theta }}_{x}$, respectively, for *x* = 1, …, *q*. Next, we solve the system of linear systems prescribed by Equation 9 for *x* = 1, …, *q*, obtaining the input image reconstructions, $p_{1}^{recon\;},\ldots ,p_{q}^{recon}$. Specifically, we use the orthogonal matching pursuit, a common CS recovery method (Tropp and Gilbert, 2007), to reconstruct the input image corresponding to each pool. Note that since the inhibitory neurons continue firing even when their pool is suppressed, we utilize only the input-output mappings for the excitatory neurons in our reconstructions. These preliminary reconstructions are combined to estimate the true percept, ***p*^recon^**, weighting them by the relative population-averaged firing rate for the excitatory neurons in each respective pool, via


$$\begin{array}{l} p^{recon\;}=\frac{m_{1E}}{\sum_{x=1}^{q}m_{xE}}p_{1}^{recon\;}+\cdots +\frac{m_{q_{E}}}{\sum_{x=1}^{q}m_{xE}}p_{q}^{recon\;}. \end{array}$$ (10)

At this point, we recollect output data throughout the next time window for the subsequent percept reconstruction. This recovery process is iterated with new data generated for each time window until the end time of the simulation is reached, producing a sequence of percept reconstructions throughout the period of perceptual multistability.

It is important to note that for time windows in which only a single pool is highly active, the relative population-averaged firing rates corresponding to excitatory neurons in the suppressed pools will be extremely small and contribute little to the final percept in Equation 10, as will be discussed further in the next section. These relative population-averaged firing rates play the role of weights that sum to one, such that the true percept is a well-defined gray-scale pixel matrix. Each weight may therefore be viewed as the current amount of evidence in favor of each percept alternative.

## 3 Results

### 3.1 Perceptual multistability dynamics in binocular rivalry model

We first consider the classical case of visual multistability in which two distinct percepts drive the dynamics of the two respective competing downstream pools of neurons, as in binocular rivalry. Utilizing the input images in Figures 1C, D and 2000 downstream neurons in each pool, we depict the evoked neuronal network dynamics in the raster plot in Figure 2B. For a simulation of 12 seconds, we plot the firing times along with the index of the firing neuron for each neuron across the two pools, demonstrating sustained spiking in both inhibitory populations and relatively high firing activity for the excitatory neurons of the dominant pool during each dominance duration. A small amount of firing activity is also observed in the excitatory neurons of the suppressed pool, especially near the time of the percept switch, which is consistent with some experimental findings (Blake and Logothetis, 2002). Overall, the raster plot demonstrates that the relative population-averaged firing rates of the excitatory neurons in each pool serve as a clear indicator of which percept is dominant and they thus effectively weight the evidence for each competing percept over a small time window.

Since balanced dynamics are exhibited by neurons in the currently dominant pool and because excitatory neurons in the suppressed pool are indeed less active due to higher inhibition than in the case of balanced dynamics, these results are indeed consistent with our model intuition and the assumptions in deriving the input-output map. Moreover, considering the inter-pool connections run from excitatory neurons in one pool to inhibitory neurons in the other pool, the inhibitory neurons in the suppressed pool continuously spike as a result of cross-pool excitation. The excitatory neurons in the suppressed pool are then made less active due to the resultant excess inhibition.

Corresponding to alternations in percept, we observe periods of rapid transition in the pool of excitatory neurons that is highly active, with only a single pool exhibiting dominance at any given point of time. As found in numerous binocular rivalry experiments (Blake and Logothetis, 2002; Wilson, 2003), the model dominance durations are irregular and generally 1 − 2.5s in length, supporting the viability of the theoretical framework. In Figure 2C, we view the rivalrous dynamics from the complementary perspective of the activity of the population-averaged dynamic firing thresholds for the excitatory and inhibitory populations of the two competing pools. We see that the thresholds of the excitatory neurons in the dominant pool increase throughout a dominance duration, whereas the thresholds of the excitatory neurons in the suppressed pool decrease. This is consistent with the notion that the increasing excitatory thresholds in the dominant pool serve to eventually reduce its activity with time. Likewise, the decreasing excitatory thresholds in the suppressed pool ultimately facilitate renewed firing activity after enough time elapses, as can be seen by the increasing number of suppressed excitatory neuron firing events in the raster plot deeper within each dominance duration.

In contrast, the thresholds of the inhibitory neurons in both pools increase at first and then decrease during each dominance period. We note that the dynamics of the inhibitory population-averaged thresholds for both pools are nearly identical. This is to be expected theoretically since inhibitory neurons in the dominant pool receive excitation from excitatory neurons in the dominant pool with connection strength $R_{IE}/\sqrt{K}$, and, considering long-range connections also have strength $R_{IE}/\sqrt{K}$, the inhibitory neurons in the suppressed pool receive steady long-range input from the excitatory neurons in the dominant pool with the exact same connection strength. The source of this excitatory input simply switches with a percept alternation, but the expected magnitude remains the same.

We note that the model exhibits a transient period in the first few hundred milliseconds, during which time the thresholds of all neurons are still relatively low and consequently alternations often occur particularly quickly. Since using data obtained during this transient period results in degraded percept reconstruction quality and potentially reflects the mixed percepts commonly reported during this initial time period in experiments (Blake et al., 1991; Pearson and Brascamp, 2008), we generally exclude the first dominance period from subsequent analysis.

Given the model dynamics agree with fundamental experimental observations, we now apply our new percept recovery framework in the context of binocular rivalry. The accuracy of each recovered percept is measured via the relative reconstruction error, ∥*p*−*p*^recon^∥/∥*p*∥. We use the Euclidean norm, $∥\text{p}∥=\sqrt{\underset{i}{\sum }p_{i}^{2}}$, and true dominant stimulus, **p**, as determined by the image input for the pool with the highest population-averaged firing rate among its excitatory neurons in a given time window.

**Figure 3:**
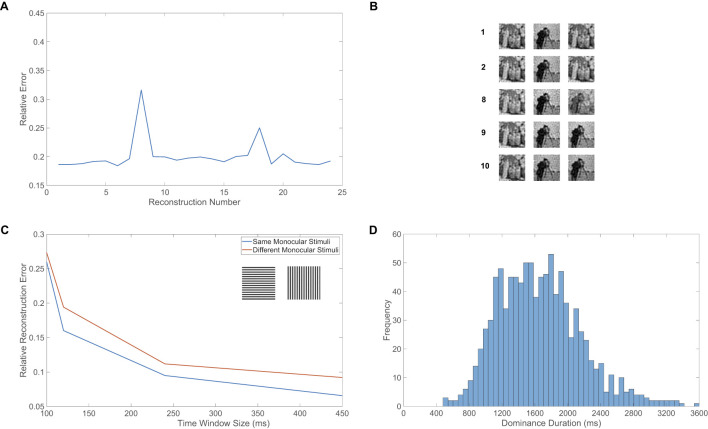
Percept recovery and alternations in the binocular rivalry model. **(A)** Relative reconstruction error for percept reconstructions obtained using downstream network dynamics collected during each respective time window of size 600ms, plotted throughout the time-course of a model simulation of time 15s. **(B)** Sample percept reconstructions for several representative labeled time windows corresponding to **(A)**, with input images as given in Figures 1C, D. Left and middle panels correspond to input reconstructions generated using the pool 1 and pool 2 input-output mappings, respectively. The right panel corresponds to the true percept reconstruction via Equation ??. **(C)** Relative reconstruction error for percepts, averaged over model simulations of time 300s, as a function of the time window size. The input images in pool 1 and 2 in the case of different monocular stimuli are given in the inset, with the corresponding reconstruction error plotted in red. In the case where the same monocular input images drive the two pools, the left image only is used, and the corresponding error is plotted in blue. **(D)** Histogram of all dominance durations across model simulations corresponding to 50 network realizations, which each have a duration of 40s, using the same two image inputs as given in the inset of **(C)**. The first time window is excluded for each simulation in this figure to account for the initial transient period in the network dynamics. The input images are normalized so that each has the same average pixel value and thus the feedforward input into each pool is comparable. All model parameters are as given in Figure 1.

The relative error for a sequence of percept reconstructions over each time window in a model simulation of length 15s is depicted in Figure 3A. The relative errors are primarily small and exhibit minor fluctuations in value. Both competing percepts are successfully recovered with similar accuracy, and sample percept reconstructions for several selected time windows are displayed in Figure 3B. We include input image reconstructions using the input-output mapping for each pool as well as the true recovered percept computed according to Equation ??. Each percept is clear and recognizable, demonstrating effective encoding via the downstream neuronal activity and relatively little information loss from the nonlinear dynamics and compressive feedforward pathway. The spikes in error for a few reconstructions are primarily attributed to time windows centered around the middle of a percept alternation, for which each pool is dominant for a non-negligible part of time within the window. For example, as seen in the reconstruction using data collected during the eighth time window, the final percept is a superposition of the two competing image inputs, typically known as a mixed percept in the context of binocular rivalry experiments (Pearson and Brascamp, 2008). This is to be expected as the relative excitatory population-averaged firing rates during this time window for pools 1 and 2 are close, with values 0.40271 and 0.59729, respectively, which is consistent with how the recovered percept more closely resembles the cameraman image driving the modestly more active pool 2. For comparison, in the first time window depicted, the relative population-averaged firing rates for the excitatory neurons in pool 1 and pool 2 are 0.99022 and 0.00978, respectively, as the collected data is entirely contained in a single dominance period.

The flexibility of this modeling and percept recovery framework analogously allows for the incorporation of binocular fusion, where, when the two monocular stimuli are sufficiently similar, a constant single percept is experienced for a length of time with no perceptual alternations. We mimic the setting of fusion by using the same input image for both pools of downstream neurons. Considering binocular fusion is crucial for depth perception and distance determination in three dimensions (Blake and Wilson, 2011), it is reasonable to expect improved percept reconstruction quality in the context of our model framework. In the case of fusion, if two pools provide evidence of the same percept, any information given by the recovered input image from the input-output mapping of the suppressed pool has the potential to improve the percept rather than diminish the reconstruction quality, as in the case of distinct competing percepts. In Figure 3C, we plot the percept recovery error averaged over all reconstructions in the entire time course of a simulation across various choices of time window size. Doing this for both the case of highly distinct monocular stimuli as in binocular rivalry, via horizontal and vertical gratings common in rivalry experiments, as well as the binocular fusion case, via identical horizontal gratings, we see that a lower percept reconstruction error is consistently achieved when the same monocular stimuli are used. In both cases, we also observe that the average reconstruction error decreases with time window size, doing so rapidly for short window sizes and then saturating once the window size is above 250ms.

Though typically only one pool in the model will be highly active at a time even in the case of identical input images, which may be a deviation from what is expected experimentally, we emphasize that the reconstructed percept will not change with time and will be of higher fidelity than in the case of highly distinct monocular inputs. We expect that the inclusion of opponency neurons that carry out dichoptic differencing of monocular inputs may allow for both pools to remain active throughout time in the case of fusion (Lansing, 1964; Bock et al., 2019). While our modeling framework does not capture three dimensional effects, we note the eventual diminishing return in reconstruction quality above this time window size is consistent with the scale of human reaction time to visual stimuli (Amano et al., 2006), and this affirms that the collection of sensory evidence for much longer amounts of time is not required to produce a reasonably accurate percept, both for fusion and rivalry.

### 3.2 Adherence of model framework to key experimental findings

Given ample evidence that humans and several nonhuman mammals demonstrate dominance duration distributions that are primarily peaked, skewed, a few seconds in length, and similar to a gamma distribution (Kovacs et al., 1996; Wilke et al., 2003), these features are common touchstones for well-grounded models of binocular rivalry. From the dynamics of the raster plot displayed in Figure 2B, we observe that the network model dominance durations are generally between 1 − 2.5s and exhibit irregularity in length, as expected from experimental findings. To further probe the dominance duration structure of the model framework, we plot in Figure 3D a histogram of dominance durations across the dynamics of 50 model network realizations, each of length 40s. Each network realization is analogous to a different individual participating in a binocular rivalry experiment, reflecting notable differences in average dominance duration across individuals in experiments (Bosten et al., 2015; Bock et al., 2019), which are likely due to genetic or chemical variations (Miller et al., 2010; Klink et al., 2010).

The histogram is peaked and right-skewed with a mean of approximately 2s, agreeing with standard experimental results. As there are generally no dominance periods less than 400ms, we see that the percept will remain fixed immediately after an alternation since adaptation in the recently suppressed pool must sufficiently decrease before another change in percept can take place. Considering the connectivity of a particular network realization does indeed impact the mean dominance duration for a simulation, analogous to how individuals are known to experience different average dominance durations, using multiple network realizations is important in order to capture the tails of the distribution. We have verified that these general features of the dominance duration distributions are robust to many other parameter choices and alternative numbers of excitatory and inhibitory neurons. The main requirements are that the dynamics of the dominant pool are balanced and the adaptation strength is moderate, such that dominance periods have a biologically realistic length and the competing pools have a marked difference in activity. Balance is expected theoretically in the large network limit across ratios of excitatory to inhibitory neurons, where *N* → ∞, *K* → ∞, and the ratio *N*_*E*_/*N*_*I*_ approaches a fixed nonzero constant. However, for finite network realizations, balanced dynamics are generally best maintained using nearly the same number of excitatory and inhibitory neurons. While for this reason our empirical results utilize a 1:1 ratio of excitatory to inhibitory neurons, analogous multistable dynamics are exhibited by the model network when using a 4:1 ratio as typical in V1 (Liu, 2004; Gilbert, 1992).

Similarly replicated in numerous experimental settings, Levelt's laws characterize how several key variations in stimulus features impact the alternation structure in binocular rivalry and reveal important facets of visual perception (Levelt, 1965; Brascamp et al., 2015). To help confirm the efficacy of our model framework, we replicate the essence of these perturbations in stimuli and compare the extent to which our model dynamics agree with the standard experimental findings. Since there is not full conformity in the notion of stimulus strength across relevant experiments, which often use different changes in the relative contrast or blur of contours in each monocular stimulus, we probe changes in stimulus strength by directly adjusting the mean feedforward drive into the two competing pools in the context of our model and keep the structure of the two image inputs the same otherwise.

**Figure 4:**
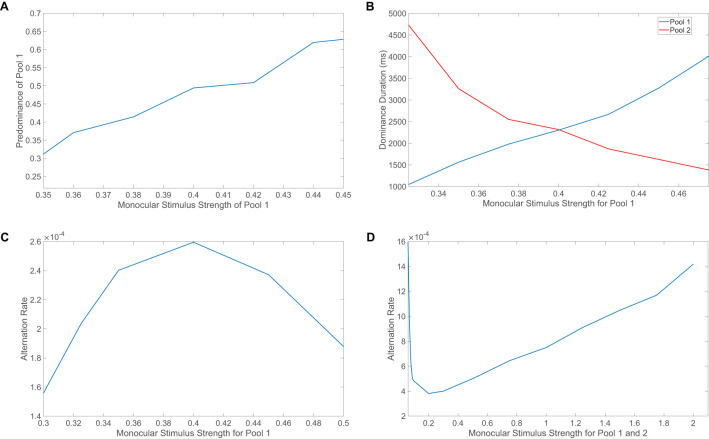
Conformance of rivalry model to Levelt's four laws. **(A)** Demonstration of Levelt's first law. The stimulus strength of pool 1 is varied and the proportion of time in which pool 1 is dominant is plotted. **(B)** Demonstration of Levelt's second law. The stimulus strength of pool 1 is varied and the average dominance durations for pool 1 (blue) and pool 2 (red) are depicted. **(C)** Demonstration of Levelt's third law. The stimulus strength of pool 1 is varied and the alternation rate is depicted in each case. For **(A–C)**, the monocular stimulus strength of pool 2 is held constant at 0.4. **(D)** Demonstration of Levelt's fourth law. The stimulus strengths of pool 1 and pool 2 are varied simultaneously and the alternation rate is plotted for each choice of stimulus strength. Note that to fairly incorporate the dominance duration corresponding to each stimulus in **(C)**, where one may be high and the other low due to the difference in stimulus strengths, we use the reciprocal of the sum of two consecutive dominance durations to compute the alternation rate. All model parameters are as given in Figure 1 and the input images are as given in the inset of Figure 3C. Each data point is the quantity averaged over 10 model realizations, each of length 40s.

Levelt's first law puts forth the trend that increasing the strength of one monocular stimulus only will increase the predominance of that monocular stimulus. Note that the predominance of a monocular stimulus refers the proportion of time that specific monocular stimulus is dominant. To test this notion, we adjust the monocular stimulus strength for the first pool only and hold the monocular stimulus strength constant for the second pool, performing a simulation for each choice of the scaled strength. The result is averaged across 10 simulations in this analysis as well as for the remaining Levelt's laws. In Figure 4A, we observe that the predominance of the first pool increases with its monocular stimulus strength around choices of strength near the fixed monocular stimulus strength for the second pool. A plausible explanation for this phenomenon is that the pool corresponding to the larger stimulus strength should generally display larger dominance durations since it is able to more strongly suppress the competing pool with its higher level of evoked activity.

Levelt's second law states that increasing the difference between the two monocular stimulus strengths will typically increase the dominance durations for the percept corresponding to the stronger of the two stimulus strengths. To test this observation, we vary the monocular stimulus strength for only the first pool and plot the resultant average dominance durations for each pool in Figure 4B. When the monocular stimulus strength for the second pool is less than that of the first pool, as the stimulus strength for the first pool is increased, the average dominance duration for the first pool increases. On the other hand, when the second pool is driven by a stronger monocular stimulus and the monocular stimulus strength for the first pool is decreased, the average dominance duration for the second pool increases as seen in the left-hand side of the plot. In each case, the difference between the stimulus strengths is increased and the higher-driven pool demonstrates an increase in dominance durations as seen in experiments.

Levelt's third law says that increasing the difference between the two monocular stimulus strengths will decrease the alternation rate. Similar to the case of Levelt's second law, in Figure 4C we adjust the monocular stimulus strength for the first pool while fixing that of the second pool, plotting the resultant alternation rate. We observe, as anticipated from experimental findings, that the fastest alternation rate is when the two monocular stimuli have equal strength, with decreasing alternation rate as the monocular stimulus strength for the first pool is perturbed. Note that since in Levelt's second law, the sum of the average dominance durations across the two pools is lowest when the monocular stimulus strengths are identical, it is to be expected that this corresponds to when the alternation rate will be highest, as predicted in Levelt's third law.

Finally, Levelt's fourth law states that increasing both monocular stimulus strengths together, such that the two strengths are equal in each case, will increase the alternation rate except at low stimulus strengths, at which the trend is reversed. To that effect, we simultaneously scale the monocular stimulus strengths of the two pools and depict the alternation rate for each choice of monocular stimulus strength in Figure 4D. We observe that the alternation rate monotonically increases for stimulus strengths above *m*_0_ = 0.2. For lower strengths, the alternation rate instead decreases as the stimulus strength increases, thus agreeing with both parts of Levelt's fourth law. The range of small stimulus strengths for which the reversal occurs is not typical in most naturalistic experiments, but several carefully-designed model-based (Shpiro et al., 2009) and experimental studies (Platonov and Goossens, 2013) give credence to this more subtle behavior for small stimulus strengths just as in the case of our model network framework.

It is worth emphasizing that Levelt's fourth law and our corresponding model dynamics provide insights into the underlying adaptation-related mechanisms for the percept alternations. For a broad range of moderate to high stimulus strengths, there is evidence that the suppressed pool is responsible for the percept switch (Brascamp et al., 2015). Decreasing the external drive strength causes longer periods of dominance since the firing thresholds for neurons in the suppressed pool generally need to decrease further in order to fire and actively escape domination; this is often referred to as the escape mechanism for the percept switch. In this case, as *m*_0_ is decreased, the suppressed pool is subject to less feedforward input drive, and fluctuations in its input current will tend to be smaller. As a result, the firing thresholds of the suppressed neurons must decrease further, over a longer span of time, for spiking activity to again be viable. It can similarly be argued that for small stimulus strengths, the dominant pool instead causes the percept switch in what is termed the release mechanism.

### 3.3 Perceptual multistability for more than two alternating percepts

Leveraging the generality of our modeling framework, we next consider perceptual multistability in the context of four alternating percepts. While our theory facilitates the analysis of alternative numbers of percepts, we choose four percepts for concreteness and to replicate the classical case of interocular grouping. We use as our image inputs the two natural scenes considered earlier in Figures 1C, D in addition to two rearranged complementary versions of the prior complete images, given in Figures 1E, F. This is to reflect experiments in which the pieces of the two presented complementary monocular stimuli may be recombined into two globally coherent images, which results in perceptual alternations between the actual monocular stimuli and the two regrouped coherent images (Kovacs et al., 1996; Suzuki and Grabowecky, 2002; Golubitsky et al., 2019; Sterzer et al., 2009). These four images drive the competitive dynamics of four corresponding downstream pools, which may be interpreted as neurons that are part of higher sensory decision-making areas that select among all reasonable percepts based on the transmitted sensory evidence (Gold and Shadlen, 2007; Li et al., 2017).

**Figure 5:**
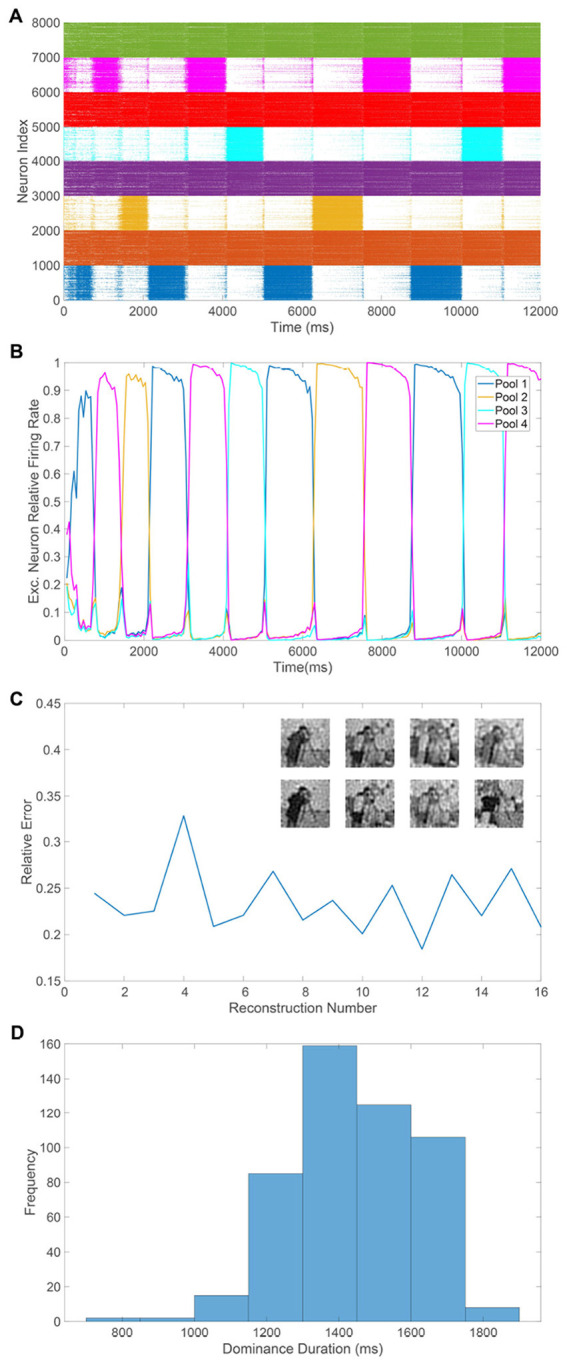
Generalization of model perceptual multistability dynamics to four percepts. **(A)** Raster plot exhibiting the firing times of downstream neurons in a model of perceptual multistability with four competing pools. Each pool contains 1, 000 excitatory neurons and 1, 000 inhibitory neurons, and distinct colors are used to distinguish among neurons in distinct populations and different pools. **(B)** Dynamics of the population-averaged excitatory neuron relative firing rates computed with Equation ?? using the same color scheme as **(A)**. **(C)** Relative reconstruction error for percept reconstructions obtained using downstream network dynamics collected during each respective time window of size 600ms, plotted throughout the time-course of a model simulation. Inset: Sample percept reconstructions for the first eight time windows. The four input images used in all panels are given in Figures 1C–F. **(D)** Histogram of all dominance durations across model simulations corresponding to 5 network realizations, which each have a duration of 160s. All model parameters are as given in Figure 1.

Utilizing 2000 neurons in each pool, the resultant model network dynamics are represented by the raster plot in Figure 5A. Similar to the case of binocular rivalry, we observe distinct blocks of time during which only the excitatory neurons of a single pool are highly active, with irregular and non-sequential switching between the dominant pools of neurons. Throughout time, we note that the inhibitory neurons in all pools are similarly active due to the long-range excitatory inputs they all receive from the dominant pool. We also observe that around the time of the percept switch, the excitatory neurons in all suppressed pools generally fire at an elevated rate, but only the excitatory neurons corresponding to the newly dominant pool ultimately persist in rapidly spiking. For the same model simulation as the raster plot, the population-averaged excitatory neuron relative firing rates, ${m_{i}}_{E}/\left(\overset{q}{\underset{x=1}{\sum }}{m_{x}}_{E}\right)$ for *i* = 1, …, 4, across the four pools are depicted in Figure 5B. These relative firing rates demonstrate clear separation between the dominant pool and the three suppressed pools, suggesting that generating a percept via the weighted combination of CS reconstructions according to excitatory neuron relative firing rates via Equation ?? is again plausible.

We gauge the encoding of percepts in the model network dynamics in Figure 5C, which shows the relative reconstruction errors generated with model data collected over 600ms time windows as used for binocular rivalry. The accuracy of the recovered percepts is relatively high and comparable to the accuracy obtained in the case of two competing pools considered earlier. Representative percepts are provided in the inset of Figure 5C, showing high fidelity reconstructions of the four possible percepts. It is important to emphasize that all four percepts are indeed realized and for roughly equal amounts of time. This is to be expected from experiments (Kovacs et al., 1996; Suzuki and Grabowecky, 2002; Golubitsky et al., 2019) and also anticipated for our model framework because the evidence for all four percepts is comparable and there is no bias in the strength of the competition between any pair of pools.

While the alternations are non-sequential and there is no sizable correlation between one percept and the next, we have verified that choosing parameters such that the adaptation dynamics are very slow will instead more likely yield sequential switching. Since in the case of three or more competing percepts, there are at least two options for the next percept, rather than one unique option as in the case of pure binocular rivalry, this gives rise to the possibility of having either sequential or non-sequential switching depending on the model parameters. In the case of four percepts, very slow adaptation makes the most recently suppressed pools unable to recover fast enough to effectively compete during the subsequent dominance duration and thus the percepts alternate sequentially in this regime.

For perceptual alternations among four percepts, the raster plot in Figure 5A depicts dominance durations primarily between 1 − 2s, which are shorter on average but of comparable magnitude to those observed in binocular rivalry. To characterize the dominance duration structure in more detail, we plot in Figure 5D a histogram of dominance durations across 5 model network realizations whose dynamics are recorded for 160s. The histogram is again peaked and skewed with a smaller mean of 1.43s, agreeing with experiments that indicate perceptual multistability with more than two percepts demonstrates moderately shorter dominance durations (Mokri et al., 2023; Riesen et al., 2019). We expect this is due to the existence of more potential options for the subsequent percept and additional overall competition in the perceptual decision-making network. It is likely that alternations among yet more percepts would result in even shorter dominance durations, but we hypothesize that in practice the brain has a limitation on the maximum number of reasonable percept options it is able to consider at any moment in time.

In contrast to our direct use of population-averaged excitatory neuron relative firing rates to determine the dominant pool, which applies to any number of competing percepts, prior work in the special case of binocular rivalry utilized a time-varying dominance metric to determine the dominant pool according to


$$\begin{array}{l} M(t)=\frac{m_{1E}(t)-m_{2E}(t)}{m_{1E}(t)+m_{2E}(t)}. \end{array}$$ (11)

This dominance metric ranged between −1 and 1, such that a value closer to 1 indicated stronger dominance of the first pool (Barkdoll et al., 2023). However, in the case of three or more pools, the existence of a real-valued function that specifically indicates both the identity of the dominant pool and the degree to which it currently dominates is unclear as is how such a complex metric could be computed by the brain. Moreover, in contrast to the methodology developed in the current work, the binocular rivalry metric in Equation 11 utilized neuronal network activity data collected over the entire dominance duration to reconstruct the percept and only the input-output mapping of the dominant pool was used. We hypothesize it is likely that the brain only leverages data collected over a time duration of the same order as reaction time to make effective and timely perceptual decisions, which would make utilizing data over such a long 1 − 2.5s time interval in the context of Equation 11 untenable. It is also worth emphasizing that the percept generated in the current work using an evidence-weighted average of potential percept options via Equation ?? allows for a unified consideration of fusion and rivalry, often yielding higher fidelity percepts in fusion due to the availability of information from multiple pools. While difficult to test experimentally, it may be the case that the percepts experienced during binocular rivalry are in fact of lower quality than those that would be experienced with just a single fused percept corresponding to only one of the rivalrous stimuli.

### 3.4 Mechanisms for perceptual alternations and amblyopia

**Figure 6:**
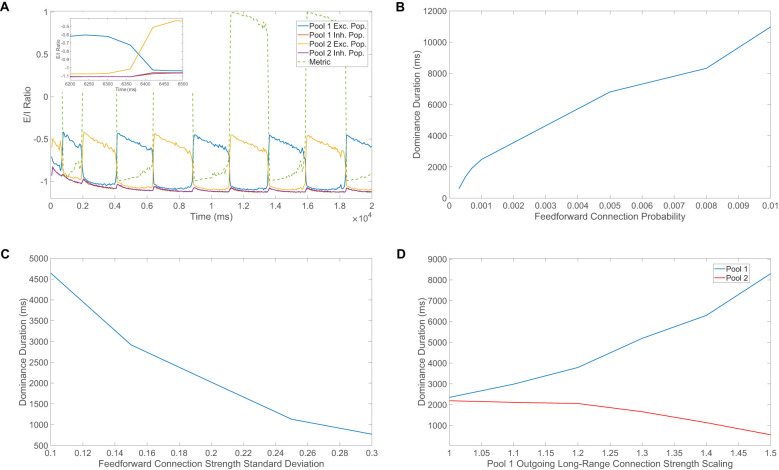
Mechanisms responsible for perceptual alternations. **(A)** Ratio of total excitatory input to total inhibitory input (E/I ratio) computed over time windows of size 60ms and averaged across neurons in each population as a function of time. Two competing pools are considered and the time-varying metric given in Equation 11 is plotted to make the identity of the dominant pool clear over fine-grained time windows. The inset depicts the E/I ratio dynamics temporally zoomed in around a single perceptual alternation. **(B)** Average dominance duration as a function of the feedforward connection probability, where the feedforward connectivity matrices have entries determined by independent identically Bernoulli distributed random variables. **(C)** Average dominance duration as a function of the standard deviation of the feedforward input across downstream neurons. In this case, for each downstream neuron, a constant homogeneous feedforward drive with the same strength as the mean in **(B)** is perturbed by an independent identically distributed normal random variable with mean 0. Note in **(B, C)**, the feedforward connection strength is scaled such that as the feedforward connection structure changes, the expected feedforward drive remains fixed at $\sqrt{K}f_{k}m_{0}$. **(D)** Average dominance duration for pool 1 (blue) and pool 2 (red) as a function of the scaling strength for the long-range connections from pool 1 to pool 2. A simulation time of 40s is used in all panels and the average dominance duration is computed over 10 realizations in **(B–D)**. All model parameters are as given in Figure 1 and the input images are given in Figures 1C, D.

While the previous sections have addressed in detail the role of adaptation and long-range competition in facilitating a perceptual alternation, our modeling framework offers a new viewpoint on the neuronal network activity around a percept switch in the context of balanced dynamics. Since the irregularity in perceptual alternations is internally generated in our modeling framework, the dynamical features are particularly indicative of what may cause such a sudden shift in the network state. In particular, in Figure 6A, we plot time dynamics of the E/I input ratios across both populations of the two competing pools in binocular rivalry. For each time bin of length 60ms, the ratio of total excitatory input to total inhibitory input is calculated and then averaged across a given population. We see that the excitatory neurons in the dominant pool primarily exhibit an E/I ratio near −1, indicative of balance. The excitatory neurons in the suppressed pool instead exhibit an E/I ratio near −0.5, demonstrating excess inhibition consistent with their lack of firing activity. As discussed previously, it is expected that the dynamics of both inhibitory populations should be similar since they receive excitatory inputs of comparable magnitude, and we note that both inhibitory populations are relatively well balanced as a result.

Shortly before the time of the percept switch, the thresholds of the excitatory neurons in the suppressed pool become sufficiently low such that more suppressed excitatory neurons begin to fire, decreasing their E/I ratio from −0.5 toward the balanced ratio of −1 and also increasing the E/I ratio of the dominant excitatory population above −1 due to extra competitive input. At the time of the switch, inhibition increases rapidly in the dominant excitatory population due to both excessively high thresholds and a cascade of firing activity among the previously suppressed competing excitatory neurons in the other pool, causing the E/I ratio of the once dominant excitatory population to jump toward −0.5. Analogously, the formerly suppressed excitatory population receives a surge of excitation due to decreased firing thresholds and less competitive input, causing a sharp drop in its E/I ratio toward −1, completing the alternation. Toward the end of a dominance period, the suppressed excitatory neurons begin to fire more rapidly and the corresponding additional long-range input into the dominant inhibitory neurons can be viewed as an increase in overall external input into the dominant inhibitory pool, which implies the extra long-range input can be absorbed into *f*_*I*_ and thus increase its value during this time. From the viewpoint of the theory given by Equation 7, the expected result is a decrease in the population-averaged firing rates for both dominant populations as *f*_*I*_ increases, which is indeed consistent with what is observed empirically and facilitates the drop in activity in the once dominant excitatory population around the time of the percept alternation. Similarly, with regard to the balance conditions given by Equation 8, if *f*_*I*_ increases too much from sufficient competitive input, the theoretically required inequality is no longer satisfied and thus balance among the dominant pool breaks down as it becomes suppressed.

To gain a complementary perspective on the role of the network connectivity structure in facilitating perceptual alternations, we also consider the impact of structured perturbations to the feedforward connectivity matrices in our model of binocular rivalry. Rather than using a spatially localized receptive field structure as in the previous sections, we instead consider feedforward connctivity matrices with independent identically distributed Bernoulli elements. Across a sequence of network simulations, we adjust the probability of a feedforward connection and compute the corresponding average dominance duration, depicting the result in Figure 6B. We see a clear trend that as the feedforward connection probablity increases, and feedforward connection density increases as a result, the mean dominance duration increases monotonically. This indicates that more homogeneous feedforward input and consequently better balanced dynamics results in a longer amount of time necessary for a perceptual alternation. Note that as the success probability for a connection is increased, we adjust the feedforward connection strength such that the expected external drive remains fixed to avoid drive strength becoming a confounding factor. As the feedforward connection probability increases, the standard deviation in the total feedforward input across downstream neurons decreases and thus the feedforward drive becomes more homogeneous. An intuitive reason for the observed relationship between feedforward connection density and dominance duration is that input fluctuations appear to play an important role in initiating the perceptual switch once the average threshold of the suppressed excitatory population becomes sufficiently small, and higher connection density and thus more homogeneity will make such switch-causing fluctuations more rare. Select neurons with more unbalanced dynamics, which result from a relatively large deviation from the mean feedforward drive, will often undergo larger fluctuations in dynamics and potentially initiate a shift in the network dynamical state.

To further demonstrate the notion that homogeneity in the network drive results in longer periods of dominance, we perturb a binocular rivalry network model with fully homogeneous feedforward input using independent identically distributed additive 0-mean normal random variable input for each downstream neuron, increasing the standard deviation across simulations. In Figure 6C, we plot the mean dominance duration as a function of the standard deviation of this perturbation, and we see that as the standard deviation increases, the average dominance duration decreases. Since the mean external drive is preserved across these network realizations, we again see that the diminishing dominance durations are a consequence of less homogeneity in the feedforward neuronal inputs and an increased likelihood of some neurons experiencing imbalanced inputs. Even when the feedforward input was fully homogeneous, perceptual alternations were still exhibited within the simulation time on some realizations, and in the case when no switches were observed, this was likely due to the simulation time being shorter than the dominance duration.

To better characterize the impact of long-range competitive connections in our network model framework and to replicate the network structure that is hypothesized to develop in amblyopia, we systematically increase the strength of all long-range connections from pool 1 to pool 2 across a sequence of network simulations. We do so using a multiplicative scaling factor, *S*_*C*_, that scales the long-range connections via $S_{C}{C_{2}^{1}}_{IE}^{ij}$ for all *i, j*. As *S*_*C*_ increases, the disparity in the strength of the long-range connections coming from the two pools increases; this is meant to reflect the suppression of information provided by the weaker eye in amblyopia, which does not have the long-range connections coming from it scaled upward in the context of our model framework. In Figure 6D, we observe that as the strength of the long-range connections from pool 1 to pool 2 increases, the mean dominance duration for pool 1 increases and the mean dominance duration for pool 2 decreases. As the same trend was observed in amblyopia experiments (Ip et al., 2021; Chow et al., 2021), we hypothesize that the brain indeed learns to increasingly give more weight to evidence from the stronger (fellow) eye to compensate for any contradictory or lower quality information from the weakened eye, and this re-weighting is potentially carried out through the same competition mechanisms as binocular rivalry. Approaches that equalize competition between monocular information or allow the brain to more robustly utilize information from the amblyopic eye are thus consistent with our model framework. This is in agreement with standard treatments, such as occlusion of the fellow eye, optical correction for the amblyopic eye, and perceptual learning therapy. These results also imply that decreasing the strength of interocular competition in the model is indeed associated with more rapid perceptual alternations, agreeing with recent experimental data analyzing the role of decreased interocular inhibition (Baker and Graf, 2009; van Loon et al., 2013; Fesi and Mendola, 2015). In general, we have verified that as the strength of the long-range connections emanating from both pools increases together, a similar increase in dominance durations occurs, but instead for both percepts. Thus, increased competition in both directions results in longer dominance durations overall.

### 3.5 Perceptual alternations give insights into potential causes of autism

Considering diagnosed cases of autism have doubled to about 2% of all children in less than twenty years (Maenner et al., 2021), better characterizing the mechanisms for autism has the potential to enhance the quality of human health and reduce the socioeconomic costs of care. The excitation/inhibition imbalance theory for autism maintains that the ratio of excitation to inhibition in the brain is elevated for individuals with autism (Rubenstein and Merzenich, 2003; Jamain et al., 2008), but further concrete evidence, especially for the precise nature of this imbalance, is still necessary. Given that several experimental studies indicate a deviation in inhibition levels may drive variations in perceptual bistability and also that individuals with increasing autism severity generally display longer periods of dominance in binocular rivalry (Robertson et al., 2013; Spiegel et al., 2019; Dieter et al., 2017), we examine the excitation/inhibition imbalance theory for autism in the context of our binocular rivalry model framework.

**Figure 7:**
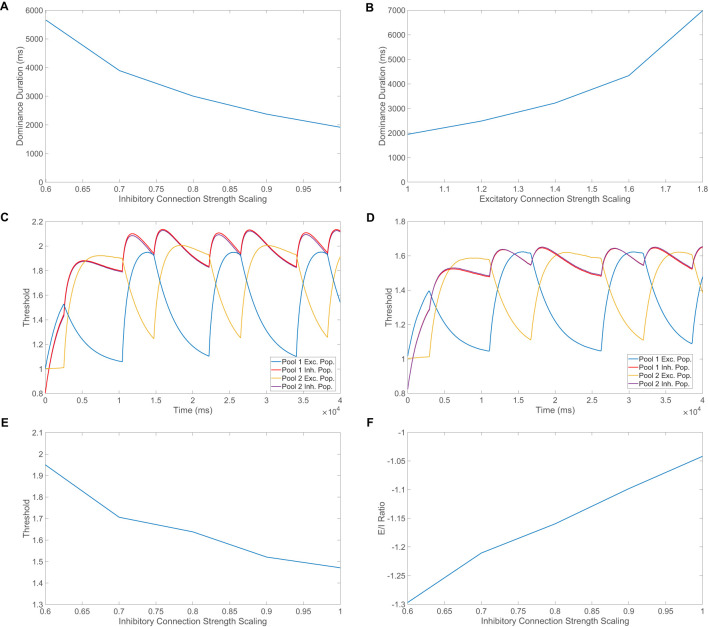
Perceptual multistability and E/I hypothesis for autism. **(A)** Mean dominance duration as a function of the recurrent inhibitory connection scaling, multiplying *R*_*II*_ and *R*_*EI*_. **(B)** Mean dominance duration as a function of the recurrent excitatory connection scaling, multiplying *R*_*EE*_ and *R*_*IE*_. **(C)** Dynamics of the population-averaged firing threshold across all 4 populations in two pools of the binocular rivalry model when all recurrent inhibitory connections are scaled by 0.6. **(D)** Dynamics of the population-averaged firing threshold across all 4 populations in two pools of the binocular rivalry model when all recurrent excitatory connections are scaled by 1.4. **(E)** Dynamic firing threshold, averaged across all excitatory neurons during their periods of dominance throughout all simulation time, as a function of the recurrent inhibitory connection scaling. **(F)** Ratio of total excitatory input to total inhibitory input across dominant excitatory neurons in each period of dominance, averaged with data collected over the entire simulation, as a function of the recurrent inhibitory connection scaling. A simulation time of 40s for two competing pools is used in all panels, and the average result is computed over 10 realizations in **(A, B)** and **(E, F)**. All model parameters are as given in Figure 1 and the input images are given in Figures 1C, D.

The magnitude of the E/I ratio in our model, |*E*/*I*|, may be elevated either by increasing the relative strength of excitatory inputs or decreasing the relative strength of inhibitory inputs; since experiments have not unanimously indicated which mechanism is at play (Gao and Penzes, 2015; Nelson and Valakh, 2015; Rosenberg et al., 2015), we examine both cases independently. First, we multiply the recurrent inhibitory connection strength parameters by a scaling factor, *S*_*I*_, such that connections from inhibitory to excitatory neurons are instead *S*_*I*_*R*_*EI*_ and connections between inhibitory neurons are *S*_*I*_*R*_*II*_. We note that since $\frac{\left|R_{EI}\right|}{\left|R_{II}\right|}=\frac{\left|S_{I}R_{EI}\right|}{\left|S_{I}R_{II}\right|}$, the balance conditions in Equation 8 remain identically satisfied for all nonzero choices of *S*_*I*_ and thus any resultant changes in network dynamics should theoretically not be a result of diminished balance. In Figure 7A, we plot the mean dominance duration as a function of the value of *S*_*I*_ used in a family of network realizations, and we observe that as the recurrent inhibitory connections become weaker, dominance durations increase in length. As decreasing *S*_*I*_ from 1 toward 0 weakens inhibition, thereby increasing the magnitude of the E/I ratio akin to more severe autism, these results are consistent with the autism-related binocular rivalry experiments that demonstrate more acute autism correlates with longer dominance durations. This clear relationship provides evidence that reduced inhibition in the brain is indeed a potential pathway to autism.

We analogously analyze the impact of instead increasing the strength of recurrent excitatory connections to elevate the magnitude of the E/I ratio in our model, multiplying all excitatory recurrent connections by a multiplicative scaling factor, *S*_*E*_. In particular, we utilize *S*_*E*_*R*_*IE*_ as the scaled strength of recurrent connections from excitatory to inhibitory neurons and *S*_*E*_*R*_*EE*_ as the scaled strength of recurrent connections among excitatory neurons. As in the case of the inhibitory connection scaling, because $\frac{\left|R_{EE}\right|}{\left|R_{IE}\right|}=\frac{\left|S_{E}R_{EE}\right|}{\left|S_{E}R_{IE}\right|}$, the balance conditions in Equation 8 remain satisfied across nonzero values of *S*_*E*_. In Figure 7B, we plot the mean dominance duration with increasing values of *S*_*E*_, and we observe that as the recurrent excitatory connections become stronger, dominance durations become longer, which again agrees with experimental results linking autism and binocular rivalry. Hence, our model underlines how either pathway toward an elevated ratio of excitation to inhibition in the brain is potentially consistent with autism and gives credence to the E/I hypothesis in general.

Our model framework also gives important new insights into why elevated |*E*/*I*| ratios may potentially cause longer dominance durations. One viewpoint is that of the threshold dynamics compared to those observed earlier in Figure 2C. We plot in Figures 7C, D the population-averaged firing thresholds for *S*_*I*_ = 0.6 and *S*_*E*_ = 1.4, respectively, reflecting representative activity corresponding to either route to an increased |*E*/*I*| ratio. An initial observation is that the thresholds of all populations peak higher than they did in the case of the default network. Second, we see that the population-averaged excitatory thresholds often reach their peak before the end of the dominance period, indicating the suppressed population is responsible for the percept switch for this drive strength as hypothesized in the release mechanism. Prior theoretical work has demonstrated that increasing spike-frequency adaptation strength up to a moderate level will generally decrease firing rates in a balanced network (Barranca et al., 2019), which agrees with the early peak in both the thresholds and firing rates of the dominant excitatory neurons, facilitating a perceptual alternation. If the thresholds peak at a higher value in either autistic limit, namely *S*_*E*_ → ∞ or *S*_*I*_ → 0, it makes intuitive sense that dominance periods become longer because the thresholds of the recently suppressed excitatory neurons must fall further to a fixed small value necessary for a potential switch. Since the strength of the long-range connections remains fixed and both inhibitory populations continue to receive matching expected excitatory drive, the average thresholds of the inhibitory populations remain close with time and generally peak shortly before those of the excitatory populations. We see on a more global level that these trends are robust for alternative choices of scaling in the autistic limit. For example, in Figure 7E, we plot the population-averaged dominant excitatory neuron firing threshold, further averaged in time over all periods of dominance, as a function of the inhibitory scaling strength, *S*_*I*_. The plotted time-averaged threshold increases as the inhibitory scaling strength becomes smaller, agreeing with the representative observation of increased thresholds seen in the time dynamics shown in Figure 7C and supporting the notion that dominance durations are increased in part due to elevated peak adaptation in autism.

As a final proof of concept for the perceptual switching mechanism and its link with autism, we analyze the E/I input ratio averaged across dominant excitatory neurons over all corresponding periods of dominance as a function of the inhibitory scaling strength in Figure 7F. As the inhibitory scaling strength decreases toward 0, in the direction of more severe autism, the average dominant excitatory neuron E/I ratio becomes more negative, starting near the balanced value of −1 for the default *S*_*I*_ = 1 and decreasing to approximately −1.3 at *S*_*I*_ = 0.6. This is indicative of excess excitation in the winning population with decreasing *S*_*I*_, likely since the expected offset in voltage given by Equation 6 becomes more positive and thus amplifies the surge of excitation around the time of the percept alternation. If the inhibition scaling is yet closer to 0, we see simulations in which no perceptual alternations occur over the timescale of the simulation. Moving instead sufficiently far away from autism for *S*_*I*_≫1, we see the opposite effect, with shorter dominance durations up to a point at which the dominant pool at any given time is unclear.

We note analogous trends are observed with increased *S*_*E*_ as described in Figures 7E, F, namely that increasing *S*_*E*_ results in higher thresholds and more negative E/I ratios among the dominant excitatory neurons, as the same underlying mechanisms are at play as when the magnitude of the *E*/*I* ratio is increased by instead decreasing *S*_*I*_. The only minor difference is that the rate of change in threshold and E/I ratio is smaller in magnitude for the *S*_*E*_ scaling since the default magnitude of the incoming recurrent inhibitory connections is larger, namely |*R*_*EI*_|>*R*_*EE*_. These results help to confirm the notion that dominance durations in perceptual multistability may provide a noninvasive measure of autism and that altering the balance of excitation and inhibition in the brain may potentially aid in treating severe autism. In fact, increasing the severity of autism via either pathway to elevating the magnitude of the E/I ratio results in monotonically increasing percept reconstruction error using our percept recovery framework, which is likely a product of longer periods of mixed percepts, as seen experimentally for individuals with autism (Robertson et al., 2013; Spiegel et al., 2019), as well as less balanced dynamics overall.

## 4 Discussion

In this work, we have developed a general neuronal network modeling framework for visual multistability that is able to incorporate an arbitrary number of competing percepts, which each take the form of realistic images. The model features pools of spiking neurons that compete via long-range connections from excitatory neurons in one pool to inhibitory neurons in the others, with an adaptive firing threshold serving as an accumulated fatigue mechanism and internally-generated irregular dynamics in the dominant pool via a balanced network architecture. We constructed a coarse-graining methodology for deriving a sequence of mappings between the input images driving the dynamics of each downstream pool and measurements of the evoked network activity of each pool, facilitating the recovery of a sequence of percept estimates that each use neuronal dynamics data collected over only a short time duration. The new percept reconstruction technique leveraged compressive sensing theory to recover the input image forcing each pool based on its evoked dynamics, and then generated a percept via a weighted combination of these preliminary reconstructions, based on the average excitatory neuron relative firing rates for each pool in a given time window. Since the model exhibited distinct blocks of time during which only a single pool of excitatory neurons was highly active, as seen in visual multistability, the dominant pool was given the largest weight in generating a percept, consistent with the potential percept for which there is the most evidence. We showed each percept was generally reconstructed with high accuracy, validating our theoretical framework, and we illustrated that the model dynamics agree with important benchmarks based on binocular rivalry experiments, exhibiting gamma-like dominance duration distributions and conforming to Levelt's laws. When the input images were instead identical, analogous to binocular fusion, we recovered a stable percept with higher reconstruction fidelity. In replicating interocular grouping, our generalized model successfully produced perceptual alternations among four reasonable percepts. Accomplishing our aim of developing a theoretical framework that unified binocular rivalry, fusion, and interocular grouping, we theorize all of these phenomena may be multistable substrates of a single dynamical system that depends on the underlying structure of the input stimuli.

With our ability to flexibly link network structure and multistable dynamics, we were able to provide a new perspective on both the network activity initiating a perceptual alternation and related psychiatric disorders. We showed that the dominant pool exhibited balanced dynamics, whereas the suppressed pools were primarily over-inhibited; near the time of the percept switch, a rapid rise in excitation in the suppressed pool, due to both lower firing thresholds and less competitive input from the weakening dominant pool, facilitated a re-balancing of dynamics that gave way to a perceptual alternation. Upon systematically creating a discrepancy in the strength of long-range competitive inputs, such that one pool sends out stronger long-range connections than the other, we observed longer dominance durations corresponding to the percept with the stronger outgoing competitive connections and we were able to establish how amblyopia might be generated by a similar competition mechanism as perceptual alternations.

We also demonstrated how more homogeneous feedforward drive and increasingly balanced dynamics result in increased dominance durations, highlighting the important role of input fluctuations in initiating a switch in percept. Since simple images are often more likely to yield homogeneous feedforward drive than natural scenes with more complicated and varied spatial structure, it is possible that this result explains why natural scenes tend to dominate simple artificial images in binocular rivalry experiments (Baker and Graf, 2009). While there was not a significant difference in the model network dynamics generated for simple and complex input image stimuli, we did find that on average the dominance durations for natural scenes were longer than those of simple images as often found experimentally (Baker and Graf, 2009; Miller, 2013; Vassall et al., 2025); in particular when one monocular stimulus was the cameraman in Figure 1C and the other was a horizontal grating as in Figure 3C, the mean dominance duration averaged over 10 realizations of length 40 seconds was 2320ms for the cameraman and 1860ms for the grating. At the same time, CS reconstructions generally only succeed for sparse stimuli (Candes et al., 2006), and thus for noisy images that are non-sparse in frequency-based domains, the fidelity of any natural scene monocular stimulus reconstruction will be higher than that of a noise stimulus, and it is likely higher-level brain areas will thus favor the natural scene. Additionally, given this line of work was among the first to use image inputs in a model-based investigation of perceptual multistability, it presents an important advance from prior work that instead used a single parameter to distinguish between inputs into competing pools rather than the gratings themselves.

While we modeled the activity of the downstream pools of neurons using an integrate-and-fire model, we expect that our theoretical framework and major findings apply to alternative single-neuron models with more detailed dynamics, such as the exponential integrate-and-fire and Hodgkin-Huxley models (Barranca et al., 2014a; Hodgkin and Huxley, 1952). Even though deriving network input-output mappings crucial to percept reconstructions is significantly more challenging in these settings, we conjecture that a means of overcoming this theoretical obstacle may be to utilize data-driven fitted input-output maps rather than analytically derived mappings (Barranca et al., 2021).

In the context of autism, we demonstrated that by elevating the ratio of excitation to inhibition in the network model, either by increasing the strength of recurrent excitatory connections or decreasing the strength of recurrent inhibitory connections, longer dominance durations were generated, as experimentally observed in individuals with increasingly severe autism. This gives credence to the E/I hypothesis for autism and indicates either pathway to imbalance is plausible. Probing the mechanism for the increase in dominance durations in our model setting, we underlined how more acute autism may be associated with increasingly high adaptation strength and strong surges in excitation at the percept switches. Since experimental evidence indicates that an imbalance in excitatory and inhibitory neuronal inputs may be implicated in schizophrenia akin to autism (Gao and Penzes, 2015; Tatti et al., 2017; Nelson and Valakh, 2015; Rosenberg et al., 2015) and individuals with bipolar disorder exhibit longer dominance durations (Miller et al., 2003), our modeling framework may provide a means of analyzing the impact of imbalanced neuronal activity in the context of other human neurological disorders and testing the viability of specific treatment options. In general, since the percept is dynamic while the stimulus is fixed, perceptual multistability is likely rooted in universal network structures in sensory systems and provides a unique viewpoint in investigating brain computations. It is possible that our framework can be similarly leveraged and extended to the study of consciousness and circuit theories for cognition, as has been done in other theoretical contexts (Koch et al., 2016; Vattikuti et al., 2016), and our model may offer additional insights due to both its realism and adaptability to conditions that are difficult to precisely control in experiments.

Since perceptual multistability is found across sensory systems (Zhou and Chen, 2009; Hupe et al., 2008; Holcombe and Seizova-Cajic, 2008), our model was purposely kept general to focus on universal organizing principles. For example, while our stimuli were grounded in vision for concreteness, we did not consider neurons with orientation selectivity (Ferster et al., 1996; Tao et al., 2006) in order to obtain mechanistic insights that apply to natural stimuli beyond simple oriented gratings. In fact, prior work demonstrates that rivalrous model dynamics still occur without receptive field structure for two competing percepts, utilizing uniformly-random sampling of input images, though the CS reconstruction quality is moderately worsened as a result (Barkdoll et al., 2023). In a similar vein, incorporating center-surround structure into the model receptive fields, akin to a standard difference of Gaussians approach (Wohrer and Kornprobst, 2009; Elder and Sachs, 2004; Lindsay, 2021), neither significantly improves the percept quality nor impacts the network multistability in our model framework (Barranca and Zhu, 2018).

We note that while it is commonly hypothesized that interocular grouping is attributed to dynamics in higher levels of the visual system (Sterzer et al., 2009; Tong, 2001), our model framework does not distinguish between phenomena that occur at different levels of the visual hierarchy and is purposely agnostic regarding eye-based vs. stimulus-based rivalry. On the one hand, this can be viewed as a limitation of the model, though on the other, the single-level modeling assumption suggests it is possible that a higher-level decision-making system is potentially responsible for moving between distinct patterns of multistable perceptual dynamics, such as binocular rivalry and interocular grouping, depending on the monocular input structure. Consistent with piecemeal rivalry (Blake et al., 1992), this type of decision-making may occur across an ensemble of spatially localized patches of the visual field, based on comparisons between small corresponding pieces of each monocular stimulus, together yielding the percept at any moment. By aggregating rivalry between many such patches in the context of a more detailed model of the visual system with deeper layers, it may be possible to produce additional phenomena that are well documented in experiments, such as how rivalry favors continuous contours, potentially due to multistability in higher areas of the visual system and feedback connections omitted from the current modeling framework (Alais et al., 2006; Alais and Melcher, 2007).

We aimed to not limit our theory to the early visual system and eye-based rivalry in particular, and rather sought to develop a broad framework for perceptual multistability that could also be viewed as a dynamical system capable of representing potential higher-level dynamics at play in making a perceptual decision, addressing the stimulus-based notion of rivalry as well. However, we expect our work may be extended to other sensory system settings by incorporating more specific structural or stimulus features. Since natural sounds and odors are known to be sparse analogous to natural images (Field, 1994; Markram et al., 1997), our CS-based percept recovery framework is likely amenable to these other contexts. On the other hand, though our perceptual multistability model is more realistic than many others since it uses as inputs pixel matrices rather than gratings or a single parameter, our current framework nevertheless treats image stimuli like two-dimensional patches rather than three-dimensional forms. Thus, an important advance would be to incorporate additional structure capable of handling depth or orientation cues in order to address other forms of visual multistability (Kornmeier and Bach, 2012). Our work also does not include top-down feedback from higher cortical areas and attention, which experiments have demonstrated to significantly modulate the features of perceptual alternations (Brown and Norcia, 1997; Zhang et al., 2011; Dieter et al., 2016; Li et al., 2017; Mokri et al., 2025). While experimental evidence indicates that long-range connections primarily stem from excitatory neurons (Stettler et al., 2002; Douglas and Martin, 2004; Tamamaki and Tomioka, 2010), we make the explicit assumption that these long-range connections specifically contact inhibitory neurons to mediate competition as has been assumed in prior modeling work on binocular rivalry (Cohen et al., 2019). It is unclear, however, which types of neurons are the primary target of long-range connections in the early visual system and whether other connectivity profiles are instead responsible for competition between pools remains an open question.

Previous theoretical studies have argued that the brain may be engineered to carry out compressive sensing reconstructions (Rozell et al., 2008) and our framework gives one potential avenue toward how CS techniques, if implementable by neuronal networks, may be leveraged in conjunction with underlying input-output mappings to generate percepts. It may still be the case that CS is not the exact recovery method utilized by our brain, and if so, it is worth underlining that many of the dynamical features and mechanisms for perceptual alternations underlined in this work are in fact agnostic to the recovery method used. It is possible that dichoptic differencing of monocular input carried out by opponency neurons (Lansing, 1964; Bock et al., 2019) plays a key role in these calculations and the identification of the dynamic percept. Including opponency neurons and feedback from later sensory layers, which could modulate the strength of competition between pools based on the difference between monocular inputs, marks an important avenue for future investigation potentially capable of explaining more subtle features of visual multistability.

## Data Availability

The original contributions presented in the study are included in the article/supplementary material, further inquiries can be directed to the corresponding author.
